# Dehydrogenative Coupling of Methanol for the Gas-Phase, One-Step Synthesis of Dimethoxymethane over Supported Copper Catalysts

**DOI:** 10.1021/acssuschemeng.0c03606

**Published:** 2020-08-06

**Authors:** Anh The To, Trenton J. Wilke, Eric Nelson, Connor P. Nash, Andrew Bartling, Evan C. Wegener, Kinga A. Unocic, Susan E. Habas, Thomas D. Foust, Daniel A. Ruddy

**Affiliations:** † National Bioenergy Center, National Renewable Energy Laboratory, 15013 Denver West Parkway, Golden, Colorado 80401, United States; ‡ Chemical Sciences and Engineering Division, Argonne National Laboratory, 9700 South Cass Avenue, Argonne, Illinois 60439, United States; § Center for Nanophase Materials Sciences, Oak Ridge National Laboratory, 1 Bethel Valley Road, Oak Ridge, Tennessee 37830, United States

**Keywords:** Oxymethylene dimethyl ethers, OMEs, Dimethoxymethane, Methanol, Dehydrogenative coupling, Supported copper catalysts

## Abstract

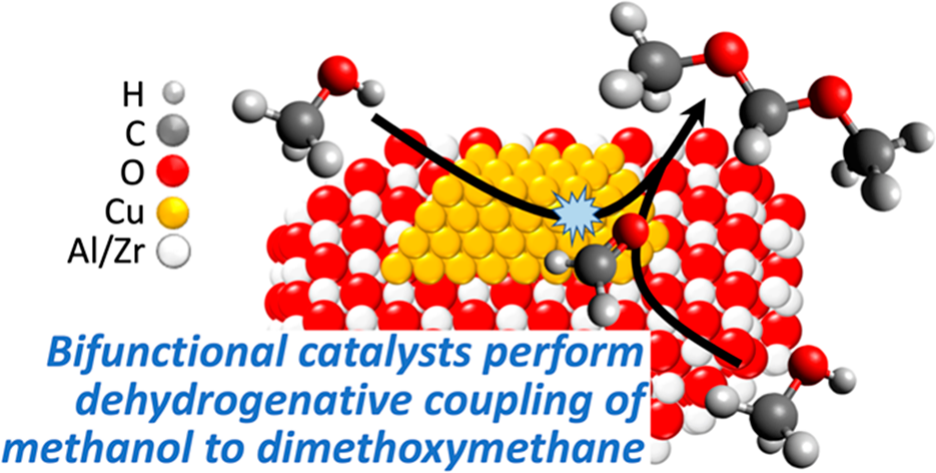

Oxymethylene dimethyl ethers (OMEs), CH_3_-(OCH_2_)_*n*_-OCH_3_, *n* = 1–5, possess attractive low-soot diesel fuel properties. Methanol is a key precursor in the production of OMEs, providing an opportunity to incorporate renewable carbon sources via gasification and methanol synthesis. The costly production of anhydrous formaldehyde in the typical process limits this option. In contrast, the direct production of OMEs via a dehydrogenative coupling (DHC) reaction, where formaldehyde is produced and consumed in a single reactor, may address this limitation. We report the gas-phase DHC reaction of methanol to dimethoxymethane (DMM), the simplest OME, with *n* = 1, over bifunctional metal–acid catalysts based on Cu. A Cu-zirconia-alumina (Cu/ZrAlO) catalyst achieved 40% of the DMM equilibrium-limited yield under remarkably mild conditions (200 °C, 1.7 atm). The performance of the Cu/ZrAlO catalyst was attributed to metallic Cu nanoparticles that enable dehydrogenation and a distribution of acid strengths on the ZrAlO support, which reduced the selectivity to dimethyl ether compared to a that obtained with a Cu/Al_2_O_3_ catalyst. The DMM formation rate of 6.1 h^–1^ compares favorably against well-studied oxidative DHC approaches over non-noble, mixed-metal oxide catalysts. The results reported here set the foundation for further development of the DHC route to OME production, rather than oxidative approaches.

## Abstract 2

Bifunctional metal−acid catalysts based on Cu set the foundation for the single-step conversion of renewable methanol to low-soot diesel fuel.

## Introduction

Diesel fuel for compression ignition (CI) engines is essential to the transportation sector, especially in mid- and heavy-duty applications, where it is advantageous over gasoline for spark ignition (SI) engines due to higher torque output and higher fuel efficiency of CI engines over SI engines.^1,2^ However, particulate matter emissions (colloquially referred to as soot) are a significant issue for CI engines. Oxygenated fuel compounds, such as dimethyl ether (DME), have demonstrated suitable diesel fuel properties and reduced sooting, leading to an ASTM International specification.^3^ However, handling gaseous DME and implementing the necessary engine modifications complicate its widespread adoption into the current infrastructure. On the other hand, oxymethylene dimethyl ethers (OMEs)—oligomeric structures of the formula CH_3_-(OCH_2_)_*n*_-OCH_3_, with *n* = 1–5—are room-temperature liquids with low vapor pressures that have received increased attention for diesel fuel applications. In addition to their excellent cetane numbers, exceeding that of conventional diesel, OMEs have demonstrated significant soot-reducing performance both as a standalone fuel and when blended into hydrocarbon diesel (e.g., yield sooting index (YSI) of 7–11 for dimethoxymethane (DMM) compared to 215 for conventional diesel fuel).^1,4−9^

The most common production route to OMEs is through a multi-step synthesis involving the acid-catalyzed acetalization reaction of methanol with formaldehyde.^4,10,11^ Anhydrous formaldehyde is preferred in this scheme to achieve high equilibrium conversion (i.e., water shifts the equilibrium away from OME products), but this is a costly step within the typical industrial oxidative dehydrogenation of methanol, where one equivalent of water is produced with each formaldehyde molecule.^12,13^ The use of methanol as the key precursor for OME production provides an attractive opportunity to utilize renewable and waste carbon sources (e.g., biomass, biogas, municipal solid waste), since gasification and methanol synthesis are established technologies.^14,15^ However, the costly production of formaldehyde, including carbon loss to DME, CO, and CO_2_ and the drying step, hinders the use of these alternative resources. Thus, routes that circumvent the carbon loss and drying cost associated with anhydrous formaldehyde production are needed to realize sustainable and cost-effective production of OMEs.

**Scheme 1:**
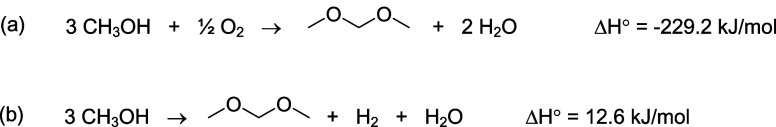
Reaction of Methanol to DMM in a Single Step via (a) Oxidative Dehydrogenation and (b) Non-oxidative Dehydrogenation

To explore new routes for OME production, the reaction of methanol to DMM, the simplest OME having *n* = 1, serves as a model reaction for catalyst development. The direct production of DMM from methanol over bifunctional catalysts, mostly via an oxidative coupling reaction, was recently reviewed.^2^ Re- and Ru-based catalysts, heteropolyacids, and various mixed oxide catalysts (e.g., V_2_O_5_–TiO_2_) have demonstrated activity for this transformation through the oxidative dehydrogenation of methanol to formaldehyde with subsequent acetalization of formaldehyde with methanol to yield DMM (Scheme 1a).^16−19^ The reaction employs molecular oxygen as the oxidant, formaldehyde is generated in situ but not isolated, and water is produced as a byproduct. Sun et al.^2^ used DMM formation rate (i.e., molar flow rate of DMM produced [mol_C_/h]/mol of metal on catalyst [mol]) to compare catalytic performance across various reports using different catalysts under different reaction conditions, and this analysis demonstrated that the majority of studies with non-noble, mixed-metal oxide catalysts had DMM formation rates in the range of 0.4–6.6 h^–1^. The most active catalysts were either supported noble metals (e.g., RuO_2_/carbon nanotube) or commercial iron-molybdate catalysts under methanol-rich feed conditions, having DMM formation rates of 23–26 h^–1^. However, the redox activity of Ru catalysts results in the formation of methyl formate (MF) with selectivity in the range of 35–70%. Hence, production of DMM using Ru catalysts would require costly and energy-intensive separation.^2^

Integrated processes to produce hydrocarbon fuels from renewable biomass are often limited in hydrogen content by the feedstock composition and require hydrogen to generate the final product (i.e., biomass molecular formula of C_*n*_H_1.5*n*_O_0.66*n*_ and fuel molecular formula of C_*n*_H_2*n*+2_).^15,20^ The oxygenated OME fuel product discussed here limits the hydrogen requirement versus hydrocarbons, but the overall H_2_ demand remains an important cost factor within a conceptual market-responsive biorefinery, where a variety of fuels and chemicals are produced to meet market demand. For example, imported H_2_ costs can range from less than $2/kg for relatively inexpensive H_2_ from steam methane reforming of natural gas to more than $4/kg for renewably sourced H_2_.^21,22^ Consequently, if H_2_ is not imported for the processes, the ability to recycle H_2_ within the biorefinery would provide a unique advantage that could reduce overall production costs. In contrast to oxidative dehydrogenation routes where hydrogen is removed as water (Scheme 1a), a dehydrogenative coupling (DHC) route to OMEs offers this H_2_-recycle option. As shown in Scheme 1b, one equivalent of water is still produced from the acetalization of methanol and formaldehyde, and gaseous H_2_ is produced as a byproduct. However, DHC approaches have been much less studied, and are limited to just a few reports for the liquid-phase conversion and no reports for the gas-phase reaction.^2^ This lack of investigation into the gas-phase reaction may be due to the difference in reaction thermodynamics (Scheme 1) or the postulated incompatibility of traditional methanol dehydrogenation at high temperatures (500–800 °C) with the acetalization reaction at low temperatures (<300 °C).^2^

We envisioned that this DHC reaction could be promoted over bifunctional metal–acid catalysts at low temperatures, similar to the oxidative pathways, where methanol dehydrogenation occurs at metallic sites with subsequent acetalization at acidic sites. For this initial investigation, Cu was chosen for the metallic functionality due to its known dehydrogenation activity and ability to be supported on a variety of acidic supports.^13,23−26^ In addition, Cu catalysts do not suffer from the Mo migration/sublimation issue with iron-molybdate oxidative dehydrogenation catalysts.^2^ Here we report the gas-phase DHC reaction of methanol to DMM over bifunctional metal–acid catalysts under the remarkably mild conditions of 200 °C and 1.7 atm. A Cu/ZrAlO catalyst demonstrated the highest activity for DMM synthesis among the four catalysts investigated, exhibiting methanol conversion of 25% with DMM selectivity of 12%. This Cu/ZrAlO catalyst was approximately 3-fold more active than Cu/Al_2_O_3_ (the second most active catalyst), achieving 40% of the DMM equilibrium-limited single-pass yield (3.0%). This greater DMM yield is attributed to decreased DME formation at the weaker acid sites of Cu/ZrAlO, as supported by pyridine-adsorption DRIFTS analysis. The DMM formation rate of 6.1 h^–1^ was achieved over Cu/ZrAlO, comparable to high-performing non-noble metal oxide catalysts in the direct DMM synthesis via methanol oxidation.^2^ This report sets the foundation for catalyst development and reaction engineering to further decrease DME formation and increase DMM selectivity via the DHC reaction.

## Results and Discussions

### Synthesis and Characterization of Supported Cu Catalysts

The following support materials were used to provide acidity for the acetalization step: commercial acidic supports SiO_2_–Al_2_O_3_ (SiAlO) and Al_2_O_3_; a non-commercial ZrO_*x*_–Al_2_O_3_ (ZrAlO) mixed oxide, comparable to a reported catalyst that exhibited high activity for OME production from methanol and formaldehyde;^27^ and a commercial ZrO_2_ for comparison to the mixed oxide. Preliminary experiments employing Cu on SiO_2_ for comparison to the SiAlO support demonstrated no activity, attributed to low acidity of SiO_2_, and were not considered for further study. Copper was supported via incipient wetness impregnation, targeting 3 wt% Cu, and elemental analysis confirmed loadings of 2.8–3.1 wt%. The Cu loadings, surface areas, acid site densities, and Brønsted:Lewis (B:L) acid site ratios are presented in Table 1. Additional physisorption data are included in Figure S1.

**Table 1:** Cu Loading, Surface Areas, Total Acid Site Density, and Brønsted:Lewis (B:L) Acid Site Ratios for Supported Cu Catalysts

catalyst	Cu loading (wt%)	surface area (m^2^/g)	acid site density (μmol/g_cat_)	B:L ratio (mol/mol)
Cu/Al_2_O_3_	3.1	120	650	0.46
Cu/SiAlO	3.1	300	1540	0.40
Cu/ZrO_2_	2.8	120	640	0
Cu/ZrAlO	3.0	110	690	0.39

**Figure 1:**
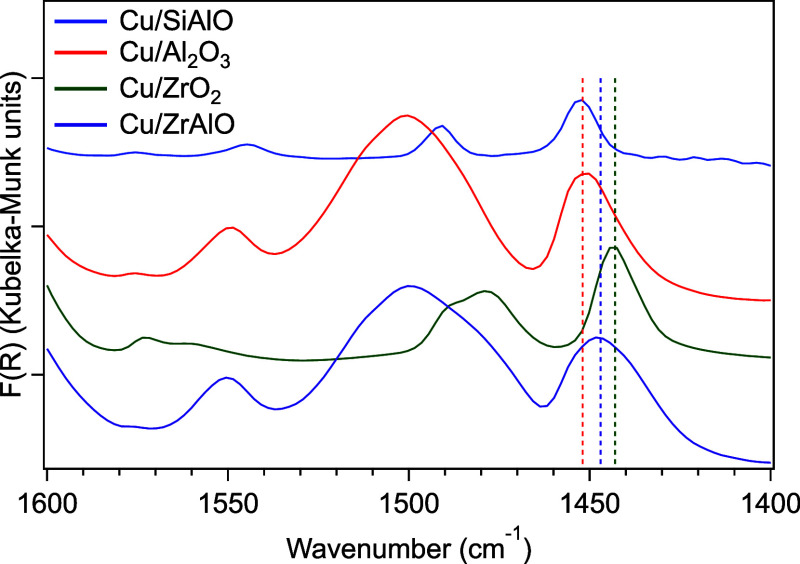
Pyridine-DRIFTS spectra for the supported Cu catalysts. The dashed lines from left to right highlight the peak centers at 1452 (Cu/Al_2_O_3_, Cu/SiAlO), 1447 (Cu/ZrAlO), and 1443 (Cu/ZrO_2_) cm^–1^.

Total acid site density was measured using temperature-programmed desorption of ammonia (Figure S2). The greater total acid site density exhibited by the Cu/SiAlO material (1540 μmol/g) is likely due to its higher surface area (300 m^2^/g vs 120 m^2^/g), whereas the other catalysts had 2-fold lower acid site densities (640–690 μmol/g). The molar ratio of B:L acid sites was measured using pyridine-adsorption DRIFTS (py-DRIFTS) after reduction with H_2_. The Cu/ZrO_2_ catalyst did not contain Brønsted sites (100% Lewis sites), and the other catalysts were predominantly Lewis-acidic, having B:L mol ratios of ca. 0.4, corresponding to ca. 70% of the total sites being Lewis acid sites. The peak position of chemisorbed pyridine near 1450 cm^–1^ is indicative of Lewis acid strength, where higher values correspond to stronger acid strength and vice versa.^28−31^ Relating changes to the 1450 cm^–1^ peak position with Lewis acid strength is analogous to relating peak positions in the 1600–1650 cm^–1^ range to Lewis acid strength, as recently demonstrated.^31^ The peak positions trended from strongest to weakest, Cu/SiAlO (1452 cm^–1^) ∼ Cu/Al_2_O_3_ (1452 cm^–1^) > Cu/ZrAlO (1447 cm^–1^) > Cu/ZrO_2_ (1443 cm^–1^) (Figure 1). The peak position observed for the Cu/ZrAlO material is indicative of a distribution of acid strengths associated with stronger Al_2_O_3_-based acid sites and weaker ZrO_*x*_-based acid sites.

A variety of techniques were employed to characterize the Cu speciation on these materials. XRD patterns of the oxidized catalysts did not exhibit reflections attributed to crystalline Cu oxides (Figure S3). These data suggest amorphous CuO_*x*_ species or small nanoparticles (<3 nm), such that broad, low-intensity reflections from the low loading of Cu species could not be detected, especially in the presence of intense reflections due to the crystalline oxide supports, Al_2_O_3_ and ZrO_2_. Previous reports have established correlations between Cu speciation and the observed reduction temperature during temperature-programmed reduction (TPR) with H_2_. The TPR profiles of the Cu materials investigated here are presented in Figure S4. Cu/SiAlO exhibited two reduction peaks similar to that observed previously with an acidic silica–alumina support,^32^ where the low temperature peak (at ca. 300 °C) represented reduction of oxo-cation-like [Cu-O-Cu]^2+^ species, and the high temperature peak (at ca. 460 °C) corresponded to small and well-dispersed CuO_*x*_ species being reduced to Cu^0^. Cu/Al_2_O_3_ exhibited a reduction peak at ca. 230 °C, similar to a previous report that attributed this to the reduction of CuO_*x*_ clusters to Cu^0^, and a broad reduction event up to 500 °C attributed to copper-aluminate-like species.^33,34^ Cu species on the reducible ZrO_2_ support reduced at a lower temperature of ca. 185 °C with a small shoulder at ca. 175 °C. Similar low-temperature reduction events were attributed to well-dispersed CuO_*x*_ species being reduced to Cu^0^.^35,36^ Addition of reducible ZrO_*x*_ species to Al_2_O_3_ at a low loading (4.79 wt% Zr) slightly lowered the reduction temperature of CuO_*x*_ species, exemplified by a peak shift to ca. 220 °C on the Cu/ZrAlO catalyst comparing to ca. 230 °C on Cu/Al_2_O_3_. In addition, there was a smaller contribution from copper-aluminate-like species, indicated by the decrease of the broad reduction event near 500 °C. Based on these TPR data, in situ reduction of the catalysts on the XRD stage at 300 °C with flowing 5% H_2_ was investigated, but no crystalline metallic Cu or Cu-oxide species were observed (Figure S3). Again, this is attributed to small particle sizes and the low Cu loading on these materials. When the Cu/SiAlO material was reduced at 500 °C, characteristic reflections for large metallic Cu particles were observed, consistent with TPR data of a high-temperature reduction event. Further, large Cu particles were observed in STEM images for Cu/SiAlO catalyst reduced at 450 °C as discussed below (Figure S5).

**Figure 2:**
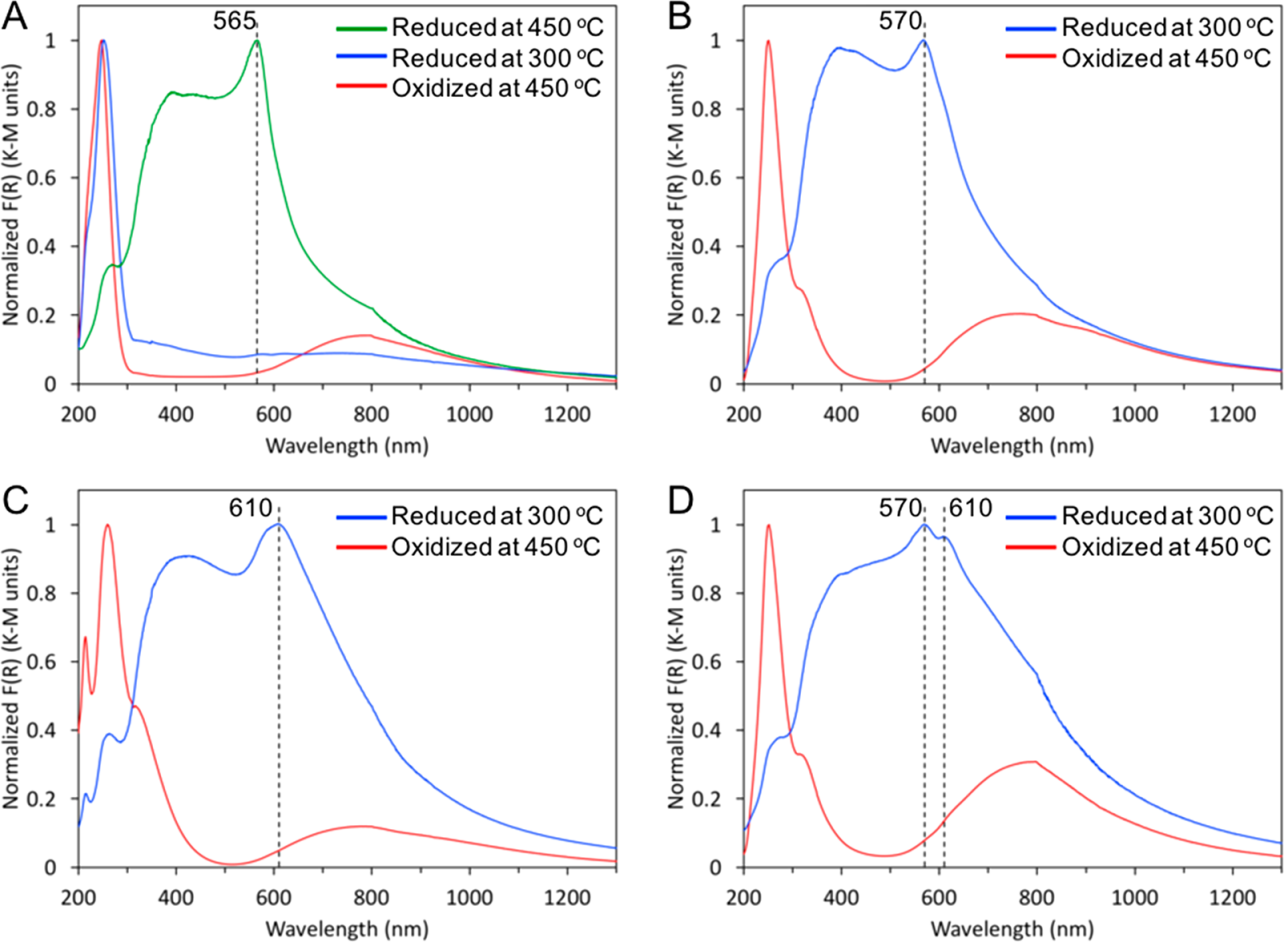
DRUV–vis–NIR spectra after oxidation at 450 °C in air and reduction at 300 °C in H_2_ for (A) Cu/SiAlO also reduced at 450 °C, (B) Cu/Al_2_O_3_, (C) Cu/ZrO_2_, and (D) Cu/ZrAlO.

DRUV–vis–NIR spectroscopy was employed to investigate Cu speciation before and after reduction, since characteristic electronic absorption spectra are well-known for Cu oxides versus metallic Cu nanoparticles.^37−40^ All of the oxidized catalysts exhibited a broad absorption in their DRUV–vis–NIR spectra centered near 800 nm and extending into the NIR, and a high-energy absorption in the UV region (Figure 2). The band at 800 nm has been attributed to both *d-d* transitions of octahedral Cu^2+^ ions and to a blue-shifted (i.e., shorter wavelength) absorption for nano-CuO versus the bulk CuO transition at ca. 870 nm, and the high-energy absorption was assigned to oxygen-to-metal charge-transfer bands in Cu oxides and clusters.^37,39,41^ Consistent with the TPR data, these features suggest Cu-oxo clusters and/or CuO nanoparticles prior to reduction. After in situ reduction at 300 °C, the spectra for all materials except Cu/SiAlO were dominated by an absorption centered between 565 and 615 nm (Figure 2B–D). This feature is characteristic for the local surface plasmon resonance (LSPR) of Cu nanoparticles.^38,40^ For Cu/SiAlO, this feature was observed after reduction at 450 °C, consistent with the TPR data (Figure 2A). The position of the LSPR is not strongly affected by Cu nanoparticle size, but it is known to be sensitive to the local electronic environment of the Cu nanoparticles.^38,42^ The LSPR peak was observed at 565–570 nm on the irreducible oxides, Cu/SiAlO and Cu/Al_2_O_3_, but was red-shifted to lower energy (610 nm) on the reducible oxide, Cu/ZrO_2_. The Cu/ZrAlO material exhibited both of these LSPR peak positions (Figure 2D), suggesting the presence of Cu nanoparticles on alumina-rich and zirconia-rich regions of the support.

**Figure 3:**
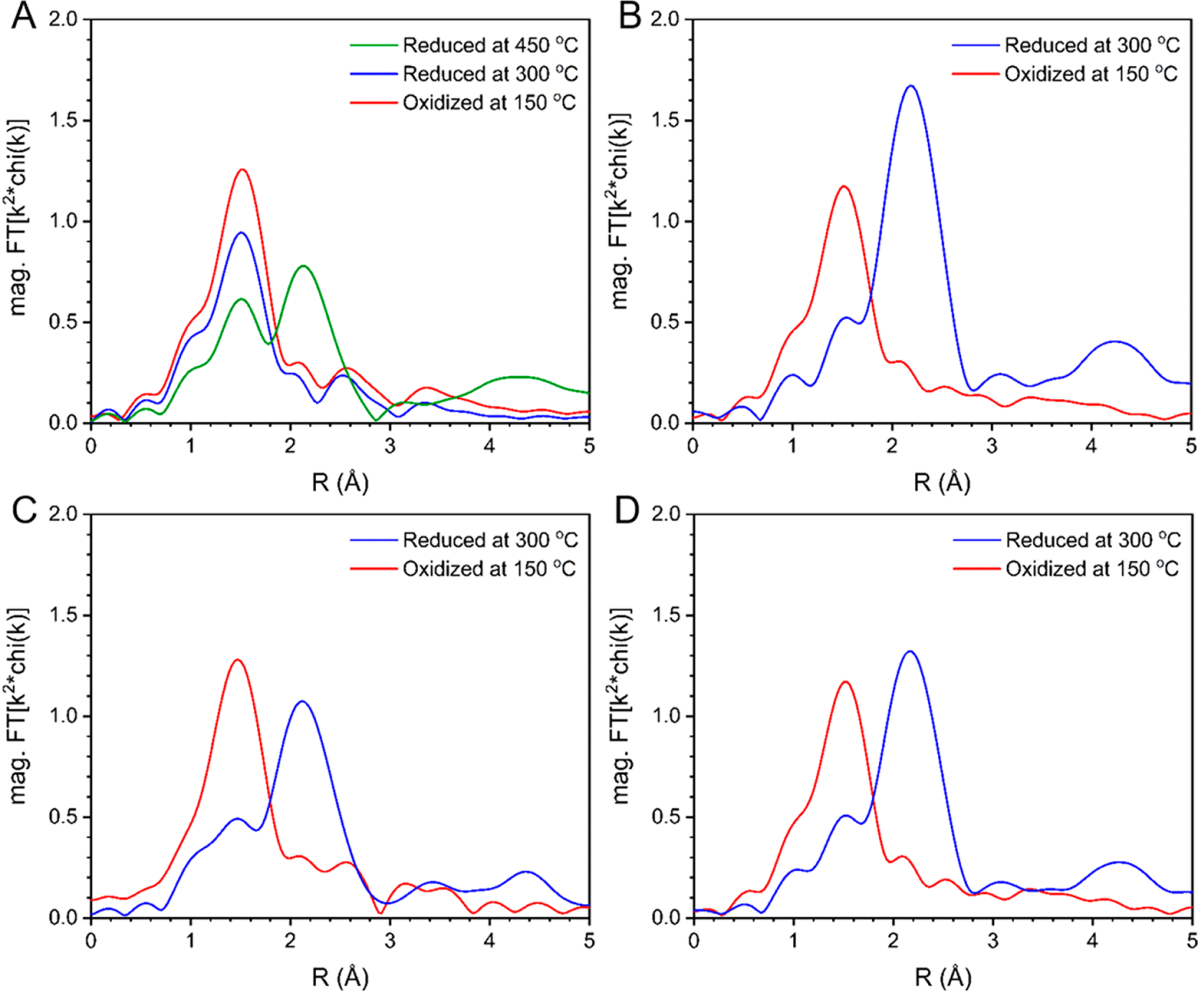
Magnitude of the Fourier transform of the Cu K edge *k*^2^-weighted EXAFS after oxidation at 150 °C in air and reduction at 300 °C in H_2_ for (A) Cu/SiAlO also reduced at 450 °C, (B) Cu/Al_2_O_3_, (C) Cu/ZrO_2_, and (D) Cu/ZrAlO.

X-ray absorption spectroscopy (XAS) was used to further characterize the Cu species. The small pre-edge peak observed at 8977.6 eV in the Cu K-edge XANES spectrum of the oxidized samples (Figure S6) indicates the presence of Cu^2+^ species prior to reduction, consistent with the assignment by DRUV–vis–NIR spectroscopy. The EXAFS analyses of the samples (Figure 3) show a single first-shell peak at ca. 1.50 Å (phase uncorrected distance) which is characteristic of Cu–O scattering. The fitted coordination numbers and bond distances (ca. 4 Cu–O bonds at 1.94 Å, Table S1) are similar to those of CuO;^43^ however, the lack of a strong second-shell Cu–Cu scattering peak (i.e., Cu–O–Cu) suggests the Cu species are highly dispersed or form small clusters, which is in agreement with the TPR and XRD results. Following reduction at 300 °C the EXAFS spectra for Cu/Al_2_O_3_, Cu/ZrO_2_, and Cu/ZrAlO exhibit contributions from both Cu–O (1.50 Å) and Cu–Cu (2.15 Å) scattering. The Cu–Cu bond distances of the three samples were determined to be 2.52 Å (Table S1), slightly shorter than bulk FCC Cu and indicative of the formation of metallic nanoparticles.^44^ The metal scattering feature was absent from the spectrum of Cu/SiAlO following reduction at 300 °C, but appeared after treatment at 450 °C in agreement with the TPR and DRUV–vis–NIR spectroscopy results.

High-angle annular dark field (HAADF) STEM imaging with energy-dispersive X-ray spectroscopy (EDS) was used to investigate the distribution of Cu in the reduced and passivated catalysts. Clustering of Cu was not readily observed in the reduced and passivated Cu/Al_2_O_3_, Cu/ZrO_2_, and Cu/ZrAlO catalysts. However, the Al-containing materials were sensitive to the electron beam and therefore were not suitable for EDS analysis at the required beam current, and the high contrast of Zr limited the ability to distinguish between oxidized Cu in the passivated samples and the Zr-containing supports by HAADF-STEM. The absence of distinct Cu clustering in these catalysts suggests that the metallic Cu particles observed by DRUV–vis–NIR spectroscopy and XAS are small, which is in agreement with the absence of crystalline metallic Cu reflections in the XRD patterns. STEM analysis of the Cu/SiAlO catalyst, on the other hand, indicated Cu clustering after reduction at both 300 and 450 °C and passivation (Figure S5). The absence of Cu–Cu metal scattering in the EXAFS spectrum and lack of LSPR feature in the DRUV–vis–NIR spectrum of the Cu/SiAlO catalyst reduced at 300 °C suggests that the clustered Cu species remained oxidized (e.g., Cu_2_O-like species) until reduction to metallic Cu at 450 °C. The combined TPR, DRUV–vis–NIR, XAS, and STEM characterization data indicate that metallic Cu nanoparticles formed on the Cu/Al_2_O_3_, Cu/ZrO_2_, and Cu/ZrAlO materials after reduction at 300 °C, and on the Cu/SiAlO after reduction at 450 °C. In combination with the acid site characterization, this suite of materials represents bifunctional metal–acid catalysts with varying acid site strength for exploration in the methanol conversion chemistry.

### Catalytic Testing of the DHC Reaction

Catalytic performance in the DHC reaction of methanol to DMM was evaluated at 1.7 atm, with varying temperature (175–225 °C) and WHSV (2.5–9.5 h^–1^). There were no effects from internal or external mass transfer limitations (calculations provided in the Supporting Information). Under these reaction conditions, the catalysts were stable and did not show considerable deactivation as measured by methanol conversion, product selectivity, or product yield over the course of 22–32 h time-on-stream (Figures S7 and S8). The results for reaction at 200 °C and WHSV of 5 h^–1^ are presented in Table 2 and compared with the results from the Cu-free support materials at the same conditions. Data is reported as the average ± standard deviation of 10–12 data points during approximately 6 h time-on-stream (Table 2). Selectivity is reported as C-selectivity. Methanol conversions were below 15% except for the most active Cu/ZrAlO catalyst, which exhibited a conversion of 24.7%. For Al_2_O_3_, ZrO_2_, and ZrAlO materials, conversion increased when Cu was added, serving as the first indication of additional reaction pathways due to the addition of metal species.

**Table 2:** Catalytic Performance of the Unmodified Supports and Supported Cu Catalysts in the Conversion of Methanol to DMM^a^

catalyst	*X* (%)	*S*_DME_ (%)	*S*_MF_ (%)	*S*_DMM_ (%)	*S*_HCs_ (%)	*S*_CO*_x_*_ (%)	H_2_ productivity (mg/g_cat_/h)
Al_2_O_3_	7.1 ± 0.1	98.3 ± 0.3	0.7 ± 0.1	0.0 ± 0.0	0.8 ± 0.1	0.2 ± 0.0	0.5
Cu/Al_2_O_3_	14.0 ± 0.2	72.3 ± 0.8	1.3 ± 0.1	7.0 ± 0.2	0.2 ± 0.0	19.3 ± 1.2	25.0
SiAlO	3.7 ± 0.5	78.1 ± 2.8	7.1 ± 1.2	0.3 ± 0.1	9.8 ± 0.8	4.7 ± 0.8	1.9
Cu/SiAlO	3.0 ± 0.1	85.7 ± 3.9	1.8 ± 0.6	3.6 ± 0.8	3.3 ± 0.7	5.6 ± 1.9	4.4
ZrO_2_	2.5 ± 0.1	2.1 ± 0.1	8.1 ± 0.1	3.2 ± 0.2	10.4 ± 0.6	76.2 ± 0.7	16.8
Cu/ZrO_2_	10.7 ± 0.2	0.5 ± 0.1	63.5 ± 1.5	0.7 ± 0.1	4.6 ± 0.1	30.7 ± 1.5	45.8
ZrAlO	9.4 ± 0.4	63.6 ± 3.0	13.5 ± 2.1	0.3 ± 0.1	3.6 ± 0.2	19.0 ± 1.0	17.8
Cu/ZrAlO	24.7 ± 0.2	47.4 ± 0.3	4.4 ± 0.1	12.0 ± 0.3	0.8 ± 0.1	35.4 ± 0.2	84.3

a Cu catalysts were reduced with H_2_ at 300 °C prior to reaction (450 °C for Cu/SiAlO). Reaction conditions: 200 °C, 1.7 atm, methanol WHSV = 5 h^–1^. *X* = conversion; *S*_*i*_ = C-selectivity of product *i*. DME = dimethyl ether, MF = methyl formate, DMM = dimethoxymethane, HCs = hydrocarbons, CO_*x*_ = CO and CO_2_.

**Scheme 2:**
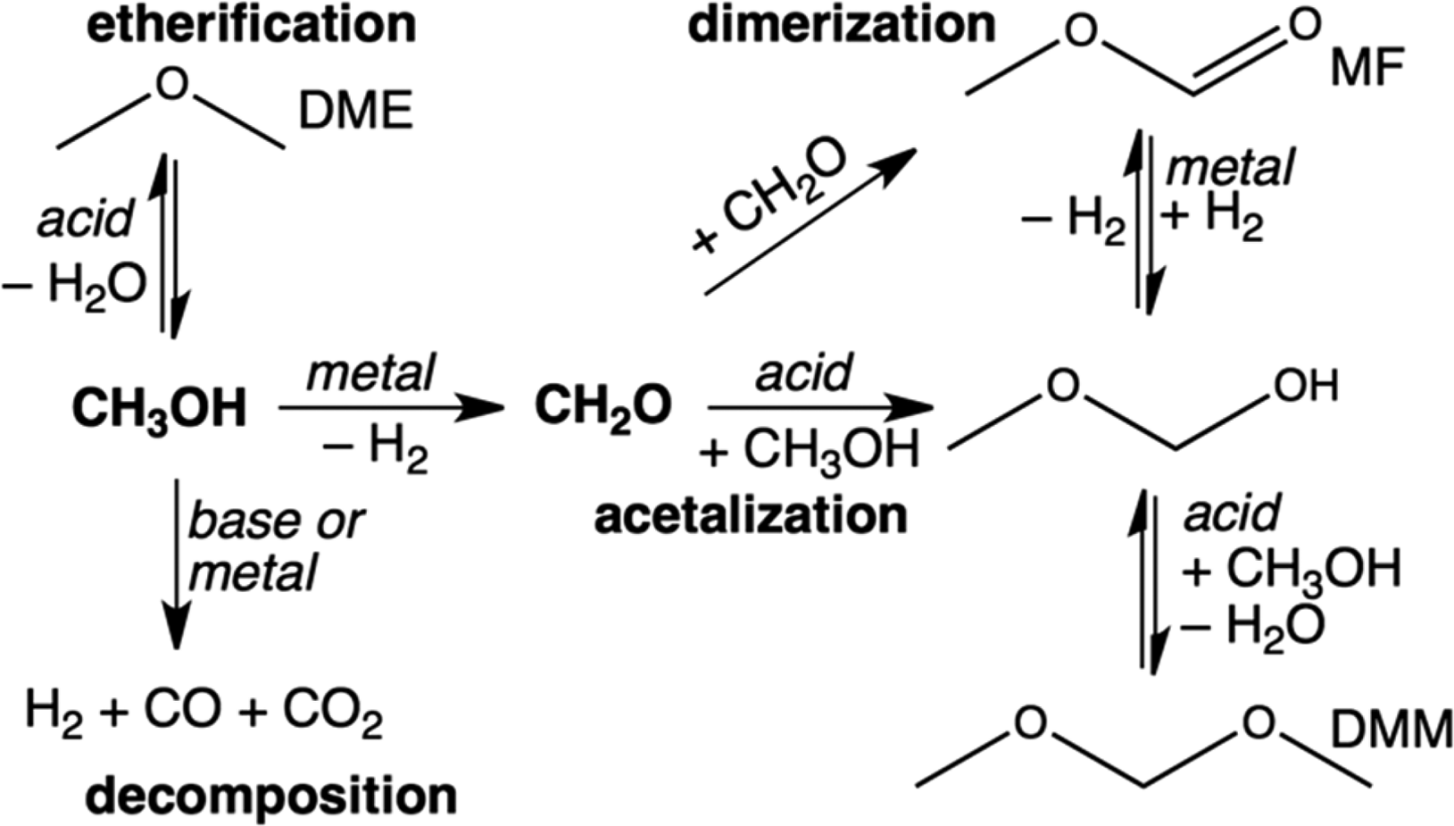
Proposed Reaction Network for DHC Reaction of Methanol to DMM over Bifunctional Metal–Acid Catalysts Metal or acid sites for each step are shown in italics.^2^

The major observed products were DME, MF, DMM, hydrocarbons (HCs, predominantly methane), CO and CO_2_ (combined as CO_*x*_), and H_2_. Formaldehyde was only observed in trace amount (less than 0.1% C-selectivity). A proposed reaction network based on the known chemistry of methanol and formaldehyde to OMEs is presented in Scheme 2,^2^ and consideration of this reaction network aids the interpretation of the observed selectivity. Acid-catalyzed dehydration of methanol to DME, which is known to occur at both Lewis and Brønsted acid sites, was the major pathway observed over the irreducible, predominantly Lewis-acidic supports, where DME C-selectivity was 98.3% and 78.1% over Al_2_O_3_ and SiAlO catalysts, respectively. The ZrO_2_ support favored methanol decomposition to CO_*x*_ (76% C-selectivity), which is often attributed to basic sites on ZrO_2_,^45,46^ and a low level of background dehydrogenation activity on this reducible support was observed (8.1% C-selectivity to MF). Dehydration was not observed at the comparatively weaker Lewis acid sites on ZrO_2_ than on Al_2_O_3_ and SiAlO catalysts. Consistent with the py-DRIFTS characterization indicating a distribution of Lewis acid strength, the ZrAlO catalyst exhibited intermediate reactivity between Al_2_O_3_ and ZrO_2_, demonstrating significantly lower C-selectivity to DME (66.7%) and CO_*x*_ (19.8%), while selectivity to the dehydrogenation product (i.e., MF) increased (13.5%).

With the addition of Cu, dehydrogenation and DHC were observed over Cu/Al_2_O_3_, Cu/ZrO_2_, and Cu/ZrAlO catalysts, evidenced by increased selectivity to MF or DMM and enhanced production of H_2_. In contrast, Cu/SiAlO exhibited slightly reduced activity compared to the support and minimal DHC activity. This is tentatively attributed to lower dehydrogenation activity of larger Cu particles observed by XRD and STEM for the Cu/SiAlO catalyst, rather than the small nano-Cu on the other catalysts. MF is the product of formaldehyde dimerization (i.e., Tischenko reaction),^47−49^ rather than participating in the DHC reaction. It is worth noting that hydrogenation of MF would enable it to participate in DMM formation, and the Cu catalysts reported here may perform this reaction with H_2_ generated via dehydrogenation. However, specific experiments to explore MF hydrogenation were not the focus of this report. The formation of DMM demonstrates the cooperativity between metal and acid sites, enabling the DHC chemistry outlined in Scheme 2. For Cu/Al_2_O_3_, the DMM C-selectivity was 7.0%, but the Al_2_O_3_-catalyzed production of DME remained the dominant pathway (72.3% C-selectivity) and methanol decomposition to CO_*x*_ was also observed (19.3% C-selectivity). Cu/ZrO_2_ significantly increased methanol conversion to MF versus the Cu-free support (63.5% versus 8.1% C-selectivity on the support). This catalyst demonstrated Cu-based dehydrogenation activity for the formation of formaldehyde with subsequent dimerization to form MF. However, the lack of DMM production is attributed to the weak Lewis acid sites on ZrO_2_ that were also inactive for dehydration. The Cu/ZrAlO catalyst, having a distribution of Lewis acid strengths compared to Al_2_O_3_ and ZrO_2_, exhibited the highest methanol conversion (24.7%) with a 12.0% C-selectivity to DMM. We attribute the decreased DME C-selectivity versus Cu/Al_2_O_3_ (47.4 vs 72.6%) to the weaker Lewis acid strength but note that there was an increase in CO_*x*_ selectivity (35.4 vs 19.3%) attributed to the ZrO_*x*_ species.

Despite the carbon-loss to CO_*x*_, the single-pass DMM carbon-yield tripled from 1.0% over Cu/Al_2_O_3_ to 3.0% over Cu/ZrAlO. Gas-phase dehydrogenation of methanol to MF over supported Cu catalysts has been reported^47−53^ but no effort has focused on the synthesis of DMM via gas-phase DHC of methanol. A few studies have investigated liquid-phase methanol dehydrogenation to DMM and although DMM selectivity in the liquid-phase products was high (i.e., close to 100%), the overall yield was either very low (below 0.5%)^54^ or not reported.^55^ Thus, the single-pass DMM yield achieved in this report (3.0%) greatly exceeds previous reports for the direct DMM synthesis from methanol via the DHC approach, and importantly, this was demonstrated at remarkably lower temperature (200 °C) than typical methanol dehydrogenation (500–800 °C).

The production of DMM through the DHC reaction is expected to be sensitive to reaction temperature due to thermodynamic equilibrium limitations. Methanol dehydrogenation is only spontaneous above 450 °C,^56^ but the coupling of formaldehyde with methanol is preferred at lower temperatures—below 300 °C for DMM^2,19,57^ and below 120 °C for OMEs^57−59^ due to equilibrium considerations. Increased reaction temperature favors the formation of formaldehyde,^56^ the key intermediate for DMM formation, and therefore the effect of reaction temperature on DMM production was investigated experimentally (Table S2). When the reaction temperature was increased from 200 to 225 °C, competing reactions from methanol dehydration to DME and decomposition to CO_*x*_ limited DMM formation. DME was the favored product over Cu on acidic catalysts (i.e., Al_2_O_3_, SiAlO and ZrAlO), and dehydrogenation activity dropped significantly, evidenced by the decrease in MF and DMM selectivity and yield (<0.5% yield). The Cu/ZrO_2_ catalyst favored MF and CO_*x*_ products (13.6 and 3.1% yield, respectively, DMM yield of 0.1%). For all catalysts, decreasing the reaction temperature from 200 to 175 °C favored dehydrogenation and DHC products over dehydration, exhibiting increased selectivity to MF and DMM and decreased selectivity to DME (Table S2). However, overall activity was also reduced significantly, resulting in low single-pass DMM yields less than 1.0%. Figure S9 presents the equilibrium carbon yield for methanol decomposition (CO), dehydrogenation (HCHO, MF), and the DHC (DMM) reaction pathways. Due to the thermodynamic limitations, the equilibrium single-pass C-yield of DMM at the present condition (200 °C, 0.85 atm of methanol) is 7.6%. Thus, the DMM C-yield over Cu/ZrAlO reported here reaches 40% of the equilibrium limit. Despite the modest equilibrium-limited yield, similar thermodynamically challenging processes remain industrially relevant (e.g., ammonia production, alkane dehydrogenation). From an economic perspective, these types of processes rely on high-value products produced in sufficiently high volumes to leverage economies of scale. On the production side, these processes typically rely on high product selectivity, efficient product separation with reactant recycle, and low residence times (i.e., rapid reaction kinetics). If demand for DMM and/or OMEs reaches that of high-volume fuel blendstocks, these same process parameters will be important in the development of the DHC reaction. From a catalyst development standpoint, reaching the equilibrium-limited yield can be addressed through tailoring the bifunctional metal–acid active sites identified here to further favor acetalization, as the marginal improvement in the DMM equilibrium yield with temperature is hindered by greater DME and CO_*x*_ equilibrium yields at higher temperatures.

**Figure 4:**
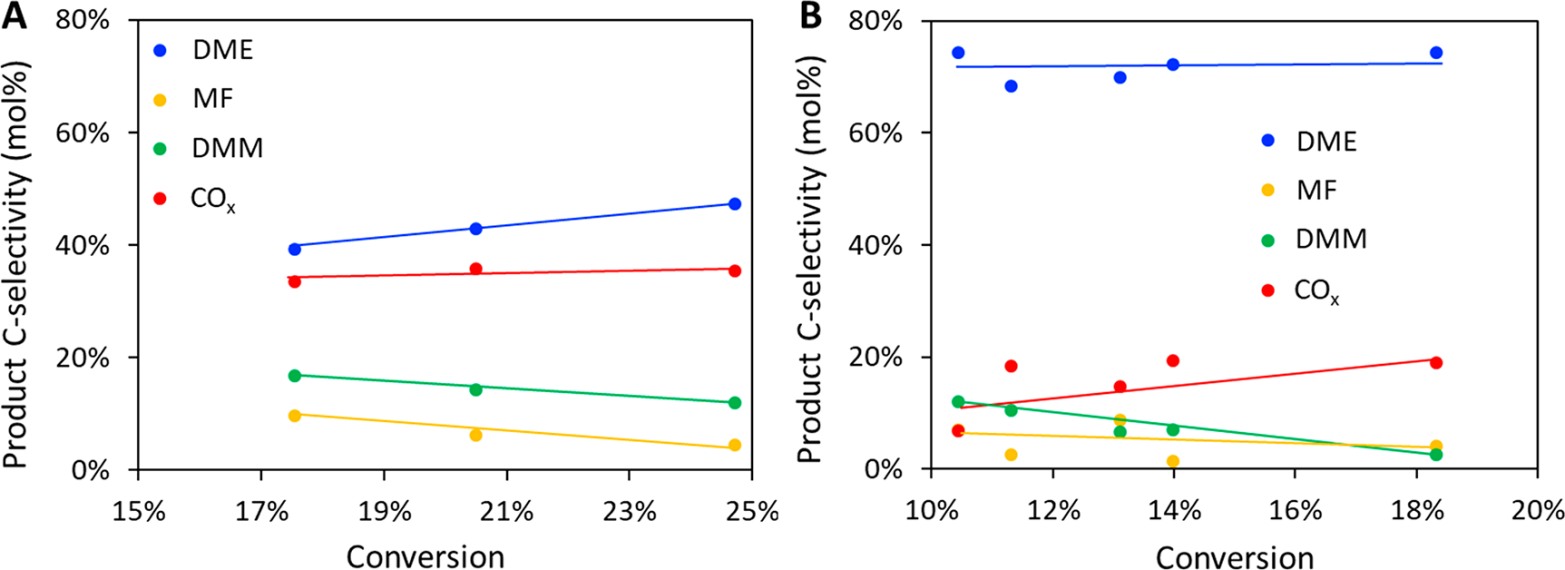
Product carbon selectivity as a function of methanol conversion over (A) Cu/ZrAlO and (B) Cu/Al_2_O_3_ catalysts. Reaction temperature was 200 °C, and reaction pressure was 1.7 atm. Lines between data points serve as a guide to the eye to highlight the trend.

The relationship between product selectivity and methanol conversion is presented in Figure 4 for Cu/ZrAlO and Cu/Al_2_O_3_ catalysts, our two best performing catalysts. Different conversions were achieved by varying WHSV from 5 to 9.5 h^–1^ for Cu/ZrAlO and from 2.5 to 5 h^–1^ for Cu/Al_2_O_3_. In general, selectivity to dehydrogenation and DHC products (i.e., MF and DMM, respectively) decreased as conversion increased, with a corresponding increase in DME or CO_*x*_ selectivity for the reaction over Cu/ZrAlO or Cu/Al_2_O_3_ catalysts, respectively. For Cu/Al_2_O_3_, high DMM selectivity was only achieved at low conversion (e.g., 12% selectivity at ca. 10% conversion) and decreased rapidly to 2.5% as conversion increased to 18%. In contrast, DMM selectivity was more consistent over Cu/ZrAlO, where it only decreased slightly with increasing conversion, from 17% at 18% conversion to 12% at 25% conversion. At similar conversions (18%), Cu/ZrAlO exhibited markedly greater selectivity to DMM than Cu/Al_2_O_3_ (16.7% vs 2.5%). It is important to note that for both catalysts, undesired products (e.g., DME and CO_*x*_) still had a significant contribution to product slates, emphasizing an area for further catalyst development.

Rather than comparing catalyst performance using turnover frequency for the multi-step, multi-site (i.e., metal and acid sites) conversion of methanol to DMM, Sun et al.^2^ compared catalysts by calculating the DMM formation rate. This metric was developed for the oxidative dehydrogenation coupling route, since this report is the first for the gas-phase DHC approach. Catalyst performance varied over a wide range of 0.4–6.6 h^–1^ for non-noble, mixed-metal oxide catalysts (e.g., V_2_O_5_/TiO_2_). The DMM formation rate observed over Cu/ZrAlO in this study was 6.1 h^–1^, which is in the range of high performing non-noble, mixed-metal oxide catalysts. The highest DMM formation rate (23.5 h^-1^) achieved for non-noble, mixed-metal oxide catalysts was over a commercial iron-molybdate catalyst, which is a well-known catalyst for formaldehyde production via methanol oxidation.^60^ Similar ranges of DMM formation rate were also reported for supported noble metal catalysts, such as Re (3–19 h^–1^) or Ru (0–26 h^–1^), but the activity varied widely depending on the support and synthesis procedure. There are significant concerns that limit applicability of these noble metal and commercial iron molybdate (FeMo) catalysts: (i) sublimation at high reaction temperature (240–300 °C) for Re and FeMo catalysts, and (ii) formation of byproduct MF (35–70% selectivity) due to the redox ability of Ru catalysts, which requires costly and energy intensive separation.^2^ Thus, the non-noble metal, supported Cu catalysts reported here, which generate DMM and hydrogen via the DHC pathway, do not contain volatile species and demonstrate lower MF selectivity, addressing these shortcomings and representing a promising foundation for continued catalyst and process development.

Finally, stability of the Cu species on the Cu/Al_2_O_3_ and Cu/ZrAlO catalysts was investigated using *in situ* EXAFS analysis (Figure S10). The spectra collected at 200 °C under flowing methanol exhibit a prominent Cu–Cu scattering peak at ca. 2.20 Å (phase uncorrected distance). At initial time-on-stream, the fitted coordination numbers of Cu/Al_2_O_3_ and Cu/ZrAlO are 9.4 and 8.2, respectively (Tables S3 and S4). As described above for the reduced Cu materials, these coordination number values are consistent with the presence of small Cu nanoparticles on the oxide supports. At longer times under flowing methanol, the intensity of the Cu–Cu scattering peak slightly increased, giving an increase in the average coordination number of less than 0.5 after 6 h for both samples, indicating minimal particle growth or sintering over this time. Although the *in situ* conditions used for XAS measurements were not able to precisely match those of the performance tests described above, the results suggest that the Cu nanoparticles are stable under these mild DHC reaction conditions. Further, these XAS results are consistent with the lack of observed catalyst deactivation during the course of reaction under varying conditions as presented above (Figures S7 and S8).

## Conclusions

Conversion of methanol to DMM serves as a model reaction for catalyst development in the single-step production of OMEs. In contrast to common oxidative dehydrogenation routes, where H_2_ is removed as water, a DHC route to OMEs generates H_2_, which can be advantageous for other processes in a biorefinery. In this report, Cu supported on commercial acidic non-reducible oxides (SiAlO and Al_2_O_3_) and acidic reducible oxides (ZrO_2_, ZrAlO) provides metal–acid bifunctionality to facilitate the gas-phase DHC reaction of methanol to DMM under remarkably mild conditions. The Cu/ZrAlO catalyst demonstrated the highest activity among the investigated catalysts, achieving a methanol conversion of 24.7% and a resulting DMM single-pass yield of 3.0%, which is 40% of the equilibrium limit under these conditions and greatly exceeds the yields of ca. 0.5% in liquid-phase chemistry. Considering the reaction network, the greater DMM yield is attributed to decreased DME formation over the lower strength acid sites of the Cu/ZrAlO catalyst. In contrast, the stronger acid sites of Cu/Al_2_O_3_ favored DME formation, and the weaker acid sites of Cu/ZrO_2_ were not active for acetalization or DME formation. These results also indicate that metallic Cu nanoparticles are effective for the dehydrogenation of methanol under these mild conditions, providing an advantage for methanol–formaldehyde coupling in a single reactor versus typical high-temperature dehydrogenation catalysts (i.e., 500–800 °C). A DMM formation rate of 6.1 h^–1^ was achieved over Cu/ZrAlO, comparable to high-performing non-noble metal-oxide catalysts for direct DMM synthesis via traditional methanol oxidation. This report sets the foundation for catalyst development to further increase DMM selectivity via tailored bifunctional metal–acid active sites that favor acetalization and disfavor decomposition, dehydration, and dimerization.

## Supporting Information Available

Experimental details; mass transfer limitation calculations; N_2_ physisorption data; plots of NH_3_-TPD, XRD, TPR, STEM, and XAS characterization data; catalytic data at varying temperatures and space velocities; plot of equilibrium-limited C-yield; and EXAFS fitting parameters with and without flowing methanol (PDF)

## References

[ref1] Liu H.; Wang Z.; Wang J.; He X.; Zheng Y.; Tang Q.; Wang J. Performance, Combustion and Emission Characteristics of a Diesel Engine Fueled with Polyoxymethylene Dimethyl Ethers (PODE3–4)/ Diesel Blends. Energy 2015, 88, 793–800. 10.1016/j.energy.2015.05.088.

[ref2] Sun R.; Delidovich I.; Palkovits R. Dimethoxymethane as a Cleaner Synthetic Fuel: Synthetic Methods, Catalysts, and Reaction Mechanism. ACS Catal. 2019, 9, 1298–1318. 10.1021/acscatal.8b04441.

[ref3] ASTM International. Standard Specification for Dimethyl Ether for Fuel Purposes; ASTM D7901-14b; West Conshohocken, PA, 2014. 10.1520/D7901-14B.

[ref4] Burger J.; Siegert M.; Ströfer E.; Hasse H. Poly(Oxymethylene) Dimethyl Ethers as Components of Tailored Diesel Fuel: Properties, Synthesis and Purification Concepts. Fuel 2010, 89 (11), 3315–3319. 10.1016/j.fuel.2010.05.014.

[ref5] Lumpp B.; Rothe D.; Pastotter C.; Lammermann R.; Jacob E. Oxymethylene Ethers as Diesel Fuel Additives of The Future. MTZ Worldwide eMag. 2011, 72 (3), 34–38. 10.1365/s38313-011-0027-z.

[ref6] Härtl M.; Seidenspinner P.; Jacob E.; Wachtmeister G. Oxygenate Screening on a Heavy-Duty Diesel Engine and Emission Characteristics of Highly Oxygenated Oxymethylene Ether Fuel OME1. Fuel 2015, 153, 328–335. 10.1016/j.fuel.2015.03.012.

[ref7] Feiling A.; Munz M.; Beidl C. Potential of the Synthetic Fuel OME1b for the Soot-free Diesel Engine. ATZextra Worldwide 2016, 21 (11), 16–21. 10.1007/s40111-015-0516-1.

[ref8] Omari A.; Heuser B.; Pischinger S. Potential of Oxymethylenether-Diesel Blends for Ultra-Low Emission Engines. Fuel 2017, 209, 232–237. 10.1016/j.fuel.2017.07.107.

[ref9] Pélerin G. K. D.; Härtl M.; Wachtmeister G. Recent Results of The Sootless Diesel Fuel Oxymethylene Ether. In Internationaler Motorenkongress 2017 Wiesbaden, June 2017; Liebl C., Beidl C., Eds.; Springer Vieweg, Wiesbaden, 2017; pp 439–456. 10.1007/978-3-658-17109-4_28.

[ref10] Hagen G. P.; Spangler M. J. Preparation of Polyoxymethylene Dimethyl Ethers by Catalytic Conversion of Dimethyl Ether with Formaldehyde Formed by Dehydrogenation of Dimethyl Ether. US 6160186 A, 2000.

[ref11] Burger J.; Ströfer E.; Hasse H. Production Process for Diesel Fuel Components Poly(Oxymethylene) Dimethyl Ethers from Methane-Based Products by Hierarchical Optimization with Varying Model Depth. Chem. Eng. Res. Des. 2013, 91 (12), 2648–2662. 10.1016/j.cherd.2013.05.023.

[ref12] Drunsel J. O.; Renner M.; Hasse H. Experimental Study and Model of Reaction Kinetics of Heterogeneously Catalyzed Methylal Synthesis. Chem. Eng. Res. Des. 2012, 90 (5), 696–703. 10.1016/j.cherd.2011.09.014.

[ref13] Lausche A. C.; Hummelshøj J. S.; Abild-Pedersen F.; Studt F.; Nørskov J. K. Application of a New Informatics Tool in Heterogeneous Catalysis: Analysis of Methanol Dehydrogenation on Transition Metal Catalysts for The Production of Anhydrous Formaldehyde. J. Catal. 2012, 291, 133–137. 10.1016/j.jcat.2012.04.017.

[ref14] Tan E. C. D.; Talmadge M.; Dutta A.; Hensley J.; Schaidle J.; Biddy M.; Humbird D.; Snowden-Swan L. J.; Ross J.; Sexton D.; Yap R.; Lukas J. Process Design and Economics for the Conversion of Lignocellulosic Biomass to Hydrocarbons via Indirect Liquefaction. Thermochemical Research Pathway to High-Octane Gasoline Blendstock Through Methanol/Dimethyl Ether Intermediates; NREL/TP-5100-62402; National Renewable Energy Lab. (NREL), Golden, CO, 2015. 10.2172/1215006

[ref15] Tan E. C.; Talmadge M.; Dutta A.; Hensley J.; Snowden-Swan L. J.; Humbird D.; Schaidle J.; Biddy M. Conceptual Process Design and Economics for the Production of High-Octane Gasoline Blendstock via Indirect Liquefaction of Biomass through Methanol/Dimethyl Ether Intermediates. Biofuels, Bioprod. Biorefin. 2016, 10 (1), 17–35. 10.1002/bbb.1611.

[ref16] Liu H.; Iglesia E. Selective One-Step Synthesis of Dimethoxymethane via Methanol or Dimethyl Ether Oxidation on H_3+n_V_n_Mo_12-n_PO_40_ Keggin Structures. J. Phys. Chem. B 2003, 107 (39), 10840–10847. 10.1021/jp0301554.

[ref17] Zhang Y.; Drake I. J.; Briggs D. N.; Bell A. T. Synthesis of Dimethyl Carbonate and Dimethoxy Methane over Cu-ZSM-5. J. Catal. 2006, 244 (2), 219–229. 10.1016/j.jcat.2006.09.002.

[ref18] Chen S.; Meng Y.; Zhao Y.; Ma X.; Gong J. Selective Oxidation of Methanol to Dimethoxymethane over Mesoporous Al-P-V-O Catalysts. AIChE J. 2013, 59 (7), 2587–2593. 10.1002/aic.14033.

[ref19] Thavornprasert K. A.; Capron M.; Jalowiecki-Duhamel L.; Dumeignil F. One-pot 1,1-Dimethoxymethane Synthesis from Methanol: a Promising Pathway over Bifunctional Catalysts. Catal. Sci. Technol. 2016, 6 (4), 958–970. 10.1039/C5CY01858G.

[ref20] Dutta A.; Schaidle J. A.; Humbird D.; Baddour F. G.; Sahir A. Conceptual Process Design and Techno-Economic Assessment of Ex Situ Catalytic Fast Pyrolysis of Biomass: A Fixed Bed Reactor Implementation Scenario for Future Feasibility. Top. Catal. 2016, 59 (1), 2–18. 10.1007/s11244-015-0500-z.

[ref21] Dillich S.; Ramsden T.; Melaina M. Hydrogen Production Cost Using Low-Cost Natural Gas. DOE Hydrogen and Fuel Cells Program Record #12024; Sept 19, 2012.

[ref22] James B. D; DeSantis D. A.; Saur G. Final Report: Hydrogen Production Pathways Cost Analysis (2013–2016); National Renewable Energy Lab. (NREL), Golden, CO, Argonne National Lab. (ANL), Argonne, IL, 2016. 10.2172/1346418

[ref23] Sato A. G.; Volanti D. P.; de Freitas I. C.; Longo E.; Bueno J. M. C. Site-selective Ethanol Conversion over Supported Copper Catalysts. Catal. Commun. 2012, 26, 122–126. 10.1016/j.catcom.2012.05.008.

[ref24] Wang Q. N.; Shi L.; Lu A. H. Highly Selective Copper Catalyst Supported on Mesoporous Carbon for the Dehydrogenation of Ethanol to Acetaldehyde. ChemCatChem 2015, 7 (18), 2846–2852. 10.1002/cctc.201500501|.

[ref25] Hernández W. Y.; De Vlieger K.; Van Der Voort P.; Verberckmoes A. Ni-Cu Hydrotalcite-Derived Mixed Oxides as Highly Selective and Stable Catalysts for the Synthesis of β-Branched Bioalcohols by the Guerbet Reaction. ChemSusChem 2016, 9 (22), 3196–3205. 10.1002/cssc.201601042. 27763728

[ref26] Ponomareva E. A.; Krasnikova I. V.; Egorova E. V.; Mishakov I. V.; Vedyagin A. A. Ethanol Dehydrogenation over Copper Supported on Carbon Macrofibers. Mendeleev Commun. 2017, 27 (2), 210–212. 10.1016/j.mencom.2017.03.035.

[ref27] Zhang J.; Fang D.; Liu D. Evaluation of Zr–Alumina in Production of Polyoxymethylene Dimethyl Ethers from Methanol and Formaldehyde: Performance Tests and Kinetic Investigations. Ind. Eng. Chem. Res. 2014, 53 (35), 13589–13597. 10.1021/ie501231a.

[ref28] Parry E. P. An Infrared Study of Pyridine Adsorbed on Acidic Solids. Characterization of Surface Acidity. J. Catal. 1963, 2 (5), 371–379. 10.1016/0021-9517(63)90102-7.

[ref29] Zaki M. I.; Hasan M. A.; Pasupulety L. Surface Reactions of Acetone on Al_2_O_3_, TiO_2_, ZrO_2_, and CeO_2_: IR Spectroscopic Assessment of Impacts of the Surface Acid-Base Properties. Langmuir 2001, 17 (3), 768–774. 10.1021/la000976p.

[ref30] Crépeau G.; Montouillout V.; Vimont A.; Mariey L.; Cseri T.; Maugé F. Nature, Structure and Strength of the Acidic Sites of Amorphous Silica Alumina: An IR and NMR Study. J. Phys. Chem. B 2006, 110 (31), 15172–15185. 10.1021/jp062252d. 16884232

[ref31] Bailleul S.; Yarulina I.; Hoffman A. E. J.; Dokania A.; Abou-Hamad E.; Chowdhury A. D.; Pieters G.; Hajek J.; De Wispelaere K.; Waroquier M.; Gascon J.; Van Speybroeck V. A Supramolecular View on the Cooperative Role of Brønsted and Lewis Acid Sites in Zeolites for Methanol Conversion. J. Am. Chem. Soc. 2019, 141 (37), 14823–14842. 10.1021/jacs.9b07484. 31464134 PMC6753656

[ref32] Bennici S.; Gervasini A.; Ravasio N.; Zaccheria F. Optimization of Tailoring of CuO_x_ Species of Silica Alumina Supported Catalysts for the Selective Catalytic Reduction of NOx. J. Phys. Chem. B 2003, 107 (22), 5168–5176. 10.1021/jp022064x.

[ref33] Yan J. Y.; Lei G. D.; Sachtler W. M. H.; Kung H. H. Deactivation of Cu/ZSM-5 Catalysts for Lean NOxReduction: Characterization of Changes of Cu State and Zeolite Support. J. Catal. 1996, 161 (1), 43–54. 10.1006/jcat.1996.0160.

[ref34] Hoang D. L.; Dang T. T. H.; Engeldinger J.; Schneider M.; Radnik J.; Richter M.; Martin A. TPR Investigations on the Reducibility of Cu Supported on Al_2_O_3_, Zeolite Y and SAPO-5. J. Solid State Chem. 2011, 184 (8), 1915–1923. 10.1016/j.jssc.2011.05.042.

[ref35] Shimokawabe M.; Asakawa H.; Takezawa N. Characterization of Copper/Zirconia Catalysts Prepared by an Impregnation Method. Appl. Catal. 1990, 59 (1), 45–58. 10.1016/S0166-9834(00)82186-7.

[ref36] Chary K. V. R.; Sagar G. V.; Srikanth C. S.; Rao V. V. Characterization and Catalytic Functionalities of Copper Oxide Catalysts Supported on Zirconia. J. Phys. Chem. B 2007, 111 (3), 543–550. 10.1021/jp063335x. 17228912

[ref37] Boccuzzi F.; Coluccia S.; Martra G.; Ravasio N. Cu/SiO_2_ and Cu/SiO_2_-TiO_2_ Catalysts: I. TEM, DR UV-Vis-NIR, and FTIR Characterization. J. Catal. 1999, 184 (2), 316–326. 10.1006/jcat.1999.2428.

[ref38] Hung L. I.; Tsung C. K.; Huang W.; Yang P. Room-Temperature Formation of Hollow Cu_2_O Nanoparticles. Adv. Mater. 2010, 22 (17), 1910–1914. 10.1002/adma.200903947. 20526993

[ref39] Giordanino F.; Vennestrøm P. N. R.; Lundegaard L. F.; Stappen F. N.; Mossin S.; Beato P.; Bordiga S.; Lamberti C. Characterization of Cu-exchanged SSZ-13: a Comparative FTIR, UV-Vis, and EPR Study with Cu-ZSM-5 and Cu-β with Similar Si/Al and Cu/Al Ratios. Dalton Trans. 2013, 42 (35), 12741–12761. 10.1039/c3dt50732g. 23842567

[ref40] Marimuthu A.; Zhang J.; Linic S. Tuning Selectivity in Propylene Epoxidation by Plasmon Mediated Photo-Switching of Cu Oxidation State. Science 2013, 339 (6127), 1590–1593. 10.1126/science.1231631. 23539599

[ref41] Pakharukova V. P.; Moroz E. M.; Kriventsov V. V.; Larina T. V.; Boronin A. I.; Dolgikh L. Y.; Strizhak P. E. Structure and State of Copper Oxide Species Supported on Yttria-Stabilized Zirconia. J. Phys. Chem. C 2009, 113 (51), 21368–21375. 10.1021/jp907652n.

[ref42] Pedersen D. B.; Wang S. Surface Plasmon Resonance Spectra of 2.8 ± 0.5 nm Diameter Copper Nanoparticles in Both Near and Far Fields. J. Phys. Chem. C 2007, 111, 17493–17499. 10.1021/jp075076x.

[ref43] Niggli P. Die Kristallstruktur einiger Oxyde I. Z. Kristallogr. - Cryst. Mater. 1922, 57, 253–299. 10.1524/zkri.1922.57.1.253.

[ref44] Otte H. M. Lattice Parameter Determinations with an X-Ray Spectrogoniometer by the Debye-Scherrer Method and the Effect of Specimen Condition. J. Appl. Phys. 1961, 32 (8), 1536–1546. 10.1063/1.1728392.

[ref45] Tatibouët J. M. Methanol Oxidation as a Catalytic Surface Probe. Appl. Catal., A 1997, 148 (2), 213–252. 10.1016/S0926-860X(96)00236-0.

[ref46] Viinikainen T.; Rönkkönen H.; Bradshaw H.; Stephenson H.; Airaksinen S.; Reinikainen M.; Simell P.; Krause O. Acidic and Basic Surface Sites of Zirconia-Based Biomass Gasification Gas Clean-Up Catalysts. Appl. Catal., A 2009, 362 (1), 169–177. 10.1016/j.apcata.2009.04.037.

[ref47] Tonner S. P.; Trimm D. L.; Wainwright M. S.; Cant N. W. Dehydrogenation of Methanol to Methyl Formate over Copper Catalysts. Ind. Eng. Chem. Prod. Res. Dev. 1984, 23 (3), 384–388. 10.1021/i300015a012.

[ref48] Cant N. W.; Tonner S. P.; Trimm D. L.; Wainwright M. S. Isotopic Labeling Studies of the Mechanism of Dehydrogenation of Methanol to Methyl Formate over Copper-Based Catalysts. J. Catal. 1985, 91 (2), 197–207. 10.1016/0021-9517(85)90334-3.

[ref49] Rodriguez-Ramos I.; Guerrero-Ruiz A.; Rojas J. L. G.; Fierro M. L. Dehydrogenation of Methanol to Methyl Formate over Copper-Containing Perovskite-Type Oxides. Appl. Catal. 1991, 68 (1), 217–228. 10.1016/S0166-9834(00)84104-4.

[ref50] Ai M. Dehydrogenation of Methanol to Methyl Formate over Copper-Based Catalysts. Appl. Catal. 1984, 11 (2), 259–270. 10.1016/S0166-9834(00)81884-9.

[ref51] Guerrero-Ruiz A.; Rodriguez-Ramos I.; Fierro J. L. G. Dehydrogenation of Methanol to Methyl Formate over Supported Copper Catalysts. Appl. Catal. 1991, 72 (1), 119–137. 10.1016/0166-9834(91)85033-R.

[ref52] Guerreiro E. D.; Gorriz O. F.; Larsen G.; Arrúa L. A. Cu/SiO_2_ Catalysts for Methanol to Methyl Formate Dehydrogenation: A Comparative Study using Different Preparation Techniques. Appl. Catal., A 2000, 204 (1), 33–48. 10.1016/S0926-860X(00)00507-X.

[ref53] Minyukova T. P.; Simentsova I. I.; Khasin A. V.; Shtertser N. V.; Baronskaya N. A.; Khassin A. A.; Yurieva T. M. Dehydrogenation of Methanol over Copper-Containing Catalysts. Appl. Catal., A 2002, 237 (1), 171–180. 10.1016/S0926-860X(02)00328-9.

[ref54] Yamakawa T.; Ohnishi T.; Shinoda S. Methanol Dehydrogenation in the Liquid Phase with Cu-Based Solid Catalysts. Catal. Lett. 1994, 23 (3–4), 395–401. 10.1007/BF00811374.

[ref55] Wu L.; Li B.; Zhao C. Direct Synthesis of Hydrogen and Dimethoxylmethane from Methanol on Copper/Silica Catalysts with Optimal Cu^+^/Cu^0^ Sites. ChemCatChem 2018, 10 (5), 1140–1147. 10.1002/cctc.201701416.

[ref56] Usachev N. Y.; Krukovsky I.; Kanaev S. A. The Nonoxidative Methanol Dehydrogenation to Formaldehyde: A review. Pet. Chem. 2004, 44, 379–394.

[ref57] Lei Y. H.; Sun Q.; Chen Z. X.; Shen J. Y. Theoretical Calculations on the Thermodynamics for the Synthesis Reactions of Polyoxymethylene Dimethyl Ethers. Acta Chim. Sinica 2009, 67 (8), 767–772.

[ref58] Schmitz N.; Burger J.; Hasse H. Reaction Kinetics of the Formation of Poly(oxymethylene) Dimethyl Ethers from Formaldehyde and Methanol in Aqueous Solutions. Ind. Eng. Chem. Res. 2015, 54 (50), 12553–12560. 10.1021/acs.iecr.5b04046.

[ref59] Schmitz N.; Homberg F.; Berje J.; Burger J.; Hasse H. Chemical Equilibrium of the Synthesis of Poly(oxymethylene) Dimethyl Ethers from Formaldehyde and Methanol in Aqueous Solutions. Ind. Eng. Chem. Res. 2015, 54 (25), 6409–6417. 10.1021/acs.iecr.5b01148.

[ref60] Gornay J.; Sécordel X.; Tesquet G.; de Ménorval B.; Cristol S.; Fongarland P.; Capron M.; Duhamel L.; Payen E.; Dubois J.-L.; Dumeignil F. Direct Conversion of Methanol Into 1,1-Dimethoxymethane: Remarkably High Productivity over an Femo Catalyst Placed Under Unusual Conditions. Green Chem. 2010, 12 (10), 1722–1725. 10.1039/c0gc00194e.

