# Supplementary material for: Dehydrogenative Coupling of Methanol for the Gas-Phase, One-Step Synthesis of Dimethoxymethane over Supported Copper Catalysts
Source: ACS Sustain Chem Eng. 2020 Aug 6;8(32):12151–60. doi: 10.1021/acssuschemeng.0c03606 (PMC10906941; doi:10.1021/acssuschemeng.0c03606)
Supplement: Supplementary file 1 — sc0c03606_si_001.pdf [file sc0c03606_si_001.pdf]

Supporting Information

Dehydrogenative Coupling of Methanol for the Gas-Phase, One-Step Synthesis of Dimethoxymethane over Supported Copper Catalysts

Anh The To,[a] Trenton J. Wilke,[a] Eric Nelson,[a] Connor P. Nash,[a] Andrew Bartling,[a] Evan C. Wegener,[b] Kinga A. Unocic,[c] Susan E. Habas,[a] Thomas D. Foust[a,*], Daniel A. Ruddy[a,*]

[a]*National Bioenergy Center, National Renewable Energy Laboratory, 15013 Denver West Parkway, Golden, CO 80401.*

[b]*Chemical Sciences and Engineering Division, Argonne National Laboratory, 9700 S. Cass Ave., Argonne, IL 60439*

[c]*Center for Nanophase Materials Sciences, Oak Ridge National Laboratory, 1 Bethel Valley Rd., Oak Ridge, TN 37830*

*corresponding authors: Thomas.Foust@nrel.gov; Dan.Ruddy@nrel.gov

Number of Pages: 23

Number of Figures: 10

Number of Tables: 4

Experimental Section

General. Commercially available catalyst supports were used as received for amorphous SiO_2-Al_2O_3 (SiAlO, Grace-Davison Davicat 3115) and gamma-Al_2O_3 (Evonik Alu130), and crushed prior to use for tetragonal-ZrO_2 (St.Gobain – NorPro SZ61152). Copper (II) nitrate hemipentahydrate ($Cu(NO_3)_2 \cdot 2.5\ H_2O$, Alfa-Aesar) and zirconyl nitrate hydrate ($ZrO(NO_3)_2 \cdot x\ H_2O$, Sigma-Aldrich) were used as received. Gas cylinders were supplied by Praxair. ACS grade methanol (>99.8%) was purchased from VWR Chemicals and used without any further purification.

Preparation of ZrAlO. The mixed oxide support was prepared similarly to Zhang et al.[1] A solution of zirconyl nitrate hydrate (0.90 g) in methanol (30 g) was added to powdered Al_2O_3 (5 g) in a plastic beaker with manual stirring. The slurry was left to dry at room temperature overnight. The material was calcined at 2 °C/min to 450 °C for 4 h with flowing air. Elemental analysis found 4.79 wt% Zr.

Incipient wetness addition of Cu. Aqueous solutions of $Cu(NO_3)_2 \cdot 2.5\ H_2O$ (0.55 g) were used to deliver ca. 3 wt% Cu to each catalyst support (4.85 g). The aqueous incipient

wetness point of each support was individually determined prior to catalyst preparation: 1.6 g/g SiAlO; 1.8 g/g Al_2O_3; 0.5 g/g ZrO_2; and 1.8 g/g ZrAlO. The materials were dried at 50 °C for 12 – 16 h, and then calcined at 2 °C/min to 450 °C for 4 h with flowing air.

Catalyst characterization. Powder X-ray diffraction (XRD) patterns were collected in the range of 20 – 80 2θ using a Rigaku Ultima IV diffractometer operating at 40 kV and 44 mA with a Cu Kα X-ray source (λ = 1.5406 Å). Powder samples (10 – 20 mg) were supported on a glass sample holder with a 0.5 mm recessed sample area and were pressed into the recession with a glass slide to obtain a uniform z-axis height. Patterns were compared to powder diffraction files (PDFs) from the International Centre for Diffraction Data (ICDD). In situ treatments were performed using a Rigaku "Reactor X" attachment. Pre-oxidized samples were supported on a black-quartz sample holder with a recessed area and pressed into the recession to achieve a uniform z-axis height. Samples were heated under flowing 10% H_2/N_2 to 300 °C (and 500 °C for Cu/SiAlO) for 2 h. XRD patterns were recorded after cooling below 50 °C with flowing 10% H_2/N_2.

Elemental analysis for Cu and Zr was determined by ICP-OES by Galbraith Laboratories (Knoxville, TN).

Surface areas were determined from N_2 physisorption isotherms at 77 K using an Autosorb-1C instrument. The samples were degassed under vacuum for 2-4 h at 250 °C prior to analysis. Surface area was determined using the Brunauer–Emmett–Teller (BET) method. Pore size distributions were determined from the adsorption data using the Barrett-Joyner-Halenda (BJH) method.

H_2 temperature programmed reduction (TPR) was performed on an Altamira AMI-390 Instrument to determine the reducibility of the supported Cu catalysts. The pre-calcined catalysts (ca. 80 mg) were loaded in a ¼" quartz U-tube reactor and held as a fixed bed on a plug of quartz wool. Samples were pretreated under 25 sccm of Argon at 10 °C/min to 500 °C for 1 h to remove adsorbed moisture during storage and cooled to 50 °C. Then, samples were reduced in 4.1% H_2/Ar flow (35 sccm) at 50 °C for 30 min, followed by heating to 500 °C at 10 °C/min with a 30 min hold. H_2 uptake was measured using a thermal conductivity detector (TCD).

Total acid site density was determined using ammonia temperature-programmed desorption (NH_3-TPD) on the same Altamira AMI-390 Instrument with gas flow rates of 25 sccm. Catalyst samples (ca. 200 mg) were reduced in flowing 4.1% H_2/Ar at 2 °C/min to 300 °C for 4 h. The samples were cooled to 120 °C in flowing He, and then saturated with flowing 10% NH_3/He for 30 min. Excess and/or physisorbed NH_3 was removed with flowing He at 120 °C for 1 h. TPD of NH_3 was performed by heating the sample from 120 to 450 °C at 30 °C/min and holding at 450 °C for 30 min. Desorbed NH_3 was measured with a TCD, and calibration was performed after each experiment by introducing 10 pulses of 10% NH_3/He through a 5 mL sample loop into a stream of flowing He.

The ratio of Brønsted to Lewis (B:L) acid sites was determined by diffuse reflectance infrared fourier transform spectroscopy of adsorbed pyridine (Py-DRIFTS) using a Thermo Nicolet iS50 FT-IR spectrometer operating at 4 cm^{-1} resolution with a Harrick praying mantis attachment and CaF_2 windows operated at ambient pressure. The Cu catalysts were reduced in 5% H_2 at 300 °C for 12 h (100 sccm, 10 °C/min ramp) and then purged with flowing Ar at 150 °C for 30 min. After pretreatment, the sample was held at

150 °C, and saturated pyridine vapor was introduced with 100 sccm of Ar for 15 min. The sample was then heated to 200 °C for 1 h under flowing 5% H_2 to remove excess and/or physisorbed pyridine, cooled to 150 °C and spectra was collected. The absorption peaks near 1545 cm^{-1} (Brønsted) and 1445 cm^{-1} (Lewis) and their relative absorption coefficients ($\varepsilon_B/\varepsilon_L$ = 0.76) were used to determine the relative B:L acid site ratios. [2, 3]

Diffuse reflectance UV-visible-NIR (DRUV-vis-NIR) spectra were collected using an Agilent Cary 5000 UV-vis-NIR spectrophotometer equipped with a Harrick Praying Mantis diffuse reflectance attachment and reaction chamber. Pre-oxidized samples (ca. 20–50 mg) were loaded and heated under flowing 10% H_2/N_2 at 10 °C/min to 300 °C for 2 h. The Cu/SiAlO sample was further reduced at 450 °C for 2 h. Spectra were collected at 50 °C in reflectance mode and MgO was used as a reference for the Kubelka-Munk function, $F(R_\infty)$.

X-ray absorption spectroscopy (XAS) measurements were performed on the bending magnet beamline of the Materials Research Collaborative Access Team (MRCAT) at the Advanced Photon Source, Argonne National Laboratory. Cu K edge spectra were

collected in transmission mode from 8720 – 9800 eV (0.35 eV steps, 0.15 seconds/step). A Cu foil spectrum was collected simultaneously during each measurement using a third ion-chamber in series for absolute energy calibration. Catalyst powders were pressed into pellets within a stainless-steel sample holder containing six wells. The sample holder was sealed in a quartz tube reactor (1” O.D., 12” length) by two Ultra-Torr fittings equipped with Kapton windows and ball valves through which gases could flow. For the oxidized and reduced samples, the reactor was heated to the treatment temperature in a clam-shell furnace under 100 sccm flow of either of 20% O_2/He or 3.5% H_2/He, held for 1 h, and then cooled to room temperature. The ball valves were then closed, isolating the samples in the treatment gases, and spectra were collected. *In situ* methanol treatments were performed using the same sample holder and reactor tube. Samples were reduced at 300 °C under 100 sccm flow of 3.5% H_2/He, then cooled to 200 °C, and the reactor was purged with He. Methanol was introduced by bubbling 50 sccm of He through a room-temperature gas saturator connected to the reactor inlet.

XAS data processing and EXAFS fitting were performed using the Demeter software suite.[4] Normalization and background subtraction were performed via standard procedures. Coordination parameters were determined from simultaneous least-squares fits in R-space of the magnitude of Fourier transform of the k^1, k^2, and k^3-weighted EXAFS. Theoretical phase shift and backscattering amplitudes were calculated using FEFF6.[5] The amplitude reduction factor (S_0^2) was determined to be 0.86 from the Cu foil and held constant in the fits of each sample.

Scanning transmission electron microscopy (STEM) was performed on a JEOL 2200FS STEM/TEM instrument equipped with a CEOS GmbH (Heidelberg, Ger) corrector on the illuminating lenses. The AMAG 5C mode was used to achieve a probe with a nominal ~150 pA current and associated resolution of a nominal 0.07 nm. The distribution of Cu, Si, Al, and O in the Cu/SiAlO catalyst was confirmed by utilizing energy dispersive X-ray spectroscopy (EDS) analysis and acquiring spectrum images with a Bruker-AXS (30mm^2 XFlash®5030 T Bruker) silicon-drift detector system (SDD) on the JEOL 2200FS STEM/TEM. Prior to analysis, the catalyst samples were reduced by heating at 5 °C/min

to 300 °C (450 °C for Cu/SiAlO) in flowing 5% H_2/N_2 (500 sccm), and holding at temperature for 2 hr. After cooling to room temperature, the catalysts were passivated in flowing 1% O_2/He (500 sccm) for 1 hr. The reduced and passivated catalysts were crushed and dispersed in hexane and deposited onto carbon-coated Mo grids for analysis.

Reaction testing. The reaction of methanol over supported Cu catalysts was evaluated in a ¼" ID fixed bed stainless steel reactor. In a typical experiment, 1.0 g of catalyst powder (particle size smaller than 300 μm) was diluted with 2.5 g of SiC (250 – 425 μm in particle size) for heat transfer enhancement. The catalyst bed was packed in between two beds of 250 – 425 μm quartz chips, with a thermocouple positioned inside the catalyst bed. The reactor temperature was controlled by a 3-zone furnace. The catalyst was pretreated under 5% H_2/95% N_2 flow (50 sccm) at 300 °C (450 °C for Cu/SiAlO) for at least 12 h. Then, the reactor was cooled to the desired reaction temperature (i.e., 175, 200 or 225 °C) under inert gas flow (5% He/95% N_2). When the temperature stabilized, reactor pressure was adjusted to 1.7 atm (i.e., 10 psig) using a back-pressure regulator, and

methanol was delivered using an HPLC pump. Carrier gas (5% He/95% N_2) flowrate and HPLC pump rate were adjusted to obtain a feed composition of 0.500/0.475/0.025 molar fraction of methanol/N_2/He, respectively, and weight hourly space velocities (WHSVs) of 2.5 – 9.5 h^{-1} based on methanol. Reactions with the Cu-free supports at similar conditions were also performed to evaluate the contribution of the support to methanol conversion. Product analysis was performed online using Agilent Technologies 7890B gas chromatographs equipped with flame ionization detectors (FID) and TCDs. The concentration of each compound was quantified by correlating its peak area with the response factor obtained from calibration standards. Sampling of the inlet stream was also performed when reactant flow was started or changed to measure concentration of the feed stream. Conversion was calculated as Σ(molar flow rate of C in all products) / (molar flow rate of inlet methanol). Product carbon yield (C-yield) was calculated as (molar flow rate of C in product *i*) / (molar flow rate of inlet methanol). Product carbon selectivity (C-selectivity) was calculated as (molar flow rate of C in product *i*) / Σ(molar flow rate of C in all products). DMM formation rate was calculated as (C-molar flowrate of DMM

produced) / (moles of Cu on catalyst). Overall C balance was in the range of 95 – 105% and H balance was in the range of 92 – 108% for all tested reactions.

Equilibrium calculations. Components for each reaction were specified as conventional components in Aspen Plus v10 and the IDEAL property method (ideal gas with Raoult's law and Henry's law) was chosen due to the low pressures of the reactions. A stream with a composition of 0.500/0.475/0.025 molar fraction of methanol, nitrogen, and helium respectively, was fed to an isothermal and isobaric REQUIL block and a sensitivity analysis was performed for each of the simulated reactions individually between 150 °C to 350 °C for Aspen to calculate the equilibrium constant (K_p) at the specified temperature using the Gibbs free energy of the reaction components. The composition of the reactor effluent was also calculated by Aspen using K_p at the specified pressure of 1.7 atm absolute, which for an ideal gas system at equilibrium is determined by solving equation $K_p = \prod_{i=products}\left(\frac{y_i P}{P_{ref}}\right) \;/\; \prod_{i=reactants}\left(\frac{y_i P}{P_{ref}}\right)$, where y_i is the mole fraction of component *i* in the gas phase, P is the total pressure (atm), and P_{ref} is the reference state pressure (1 atm). With the composition of the reactor effluent known, product C-yield was calculated

across the reactor for each individually simulated reaction by (molar flow rate of C in product *i*) / (molar flow rate of inlet methanol).

Mass transfer limitation assessment.

External mass transfer limitation for the reaction was evaluated using Mears criterion.[6]

The observed reaction rate over the Cu/ZrAlO catalyst under typical conditions (200 °C 1.7 atm, and 5 h^{-1}) was 0.011 (mol/kg-cat/s), giving a calculated MR value of 0.007, which is much less than 0.15, indicating the lack of external mass transfer limitation. Details of the calculation are presented here:

$MR = \frac{-r'_A(obs) * \rho_b * R}{k_c C_b}$, where

$-r'_A(obs)$: observed reaction rate (mol/kg cat/s)

R : catalyst particle radius, 1.5 x10^{-4} (m)

ρ_b : bulk density of catalyst bed, $\rho_b = (1 - \Phi)*\rho_c$, where

Φ: porosity of catalyst bed = 0.4

ρ_c: solid density of catalyst pellet, 3950 kg/m^3

C_b : bulk reactant concentration, 21.9 (mol/m^3)

k_C : mass transfer coefficient (m/s), was calculated from mass transfer correlation in packed bed reactor:[7]

Φ*Sh = 0.453*$Re^{1.453}$*$Sc^{1/3}$, where

Re is Reynolds number, ratio of dynamic forces to viscous forces

$Re = \frac{U * d_p}{\upsilon}$, with

U: linear velocity of stream, calculated from volumetric flowrate of reaction mixture (0.0066 m^3/h) at reaction conditions (200 °C and 1.7 atm). U = 0.058 m/s

d_p : catalyst partical diameter, $3*10^{-4}$ (m)

υ : kinematic viscosity of stream, estimated for N_2 at 200 °C and 1.7 atm. $\upsilon = 3*10^{-5}$ (m^2/s)

⇨ Re = 0.58 (indicating that the reactant flow through catalyst bed was in the laminar regime)

Sc is Schmidt number, ratio of the viscosity to molecular diffusion rate

$Sc = \frac{\upsilon}{D_{AB}}$, with

D_{AB} : gas phase diffusivity, typical value for bulk gas (10^{-5} m^2/s) was used

⇨ Sc = 3

Sh is Sherwood number, ratio of a convective mass transfer rate to a diffusion rate

$$Sh = \frac{k_C * d_p}{D_{AB}} = 0.74 \text{ (calculated from mass transfer correlation)}$$

➔ Calculated mass transfer coefficient was 0.025 m/s

Internal mass transfer limitation was evaluated using Weisz-Prater criterion.[6] The calculated C_{WP} was 0.041, which is much less than 1, indicating a lack of internal mass transfer limitation. Details of the calculation are presented here:

$$C_{WP} = \frac{-r'_A(obs) * \rho_c * R^2}{D_e C_s}$$, where

C_s : surface reactant concentration, assuming equal C_b as external transfer limitation was negligible as shown above

D_e : effective diffusivity, m^2/s, calculated as

$D_e = D_{AB}\frac{\Phi * \sigma_c}{\tau}$, where common values of below parameters were obtained from [6]

σ_c : constriction factor = 0.8

τ : tortuosity = 3.0

⇨ $D_e = 0.11 \times 10^{-5}$ m^2/s

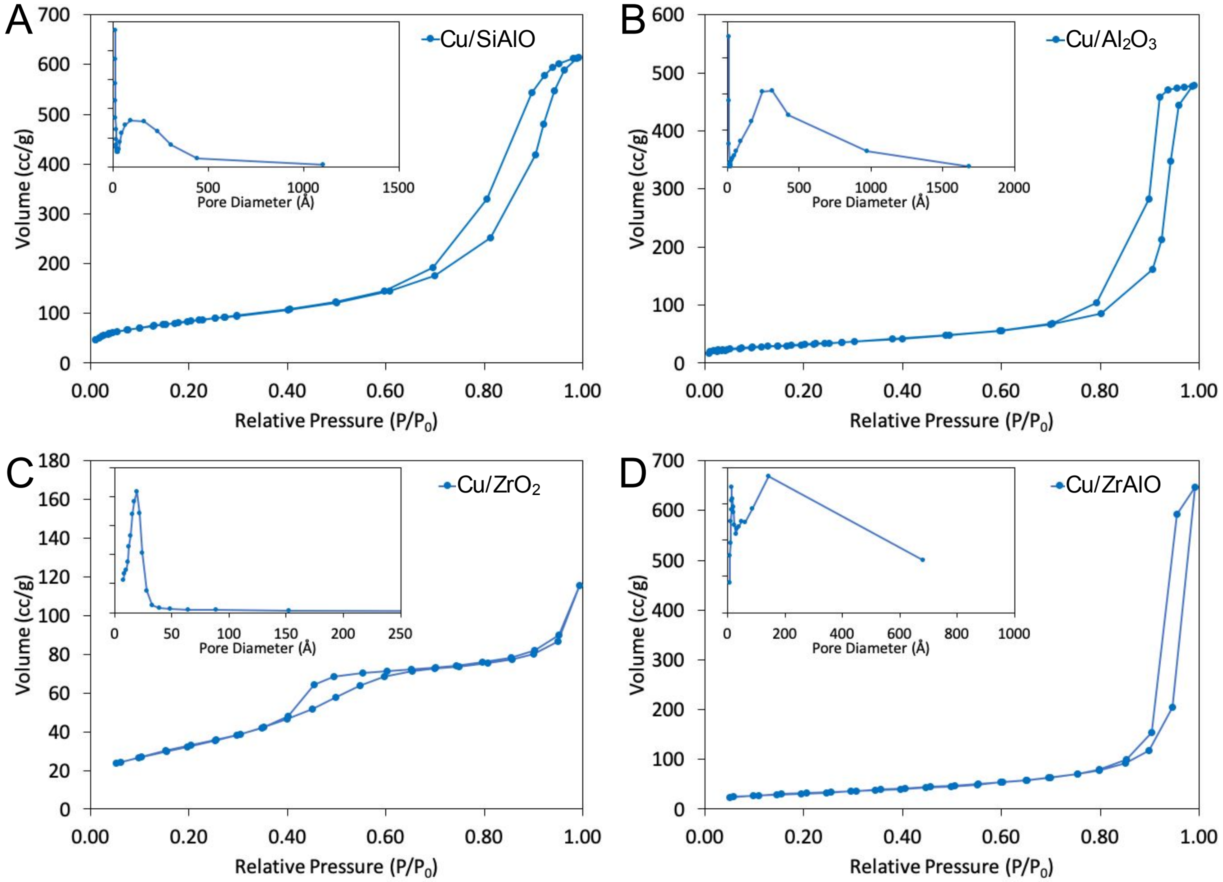

Figure S1. N_2 physisorption isotherms and pore size distributions (insets) for supported Cu catalysts (A) Cu/SiAlO, (B) Cu/Al_2O_3, (C) Cu/ZrO_2 and (D) Cu/ZrAlO.

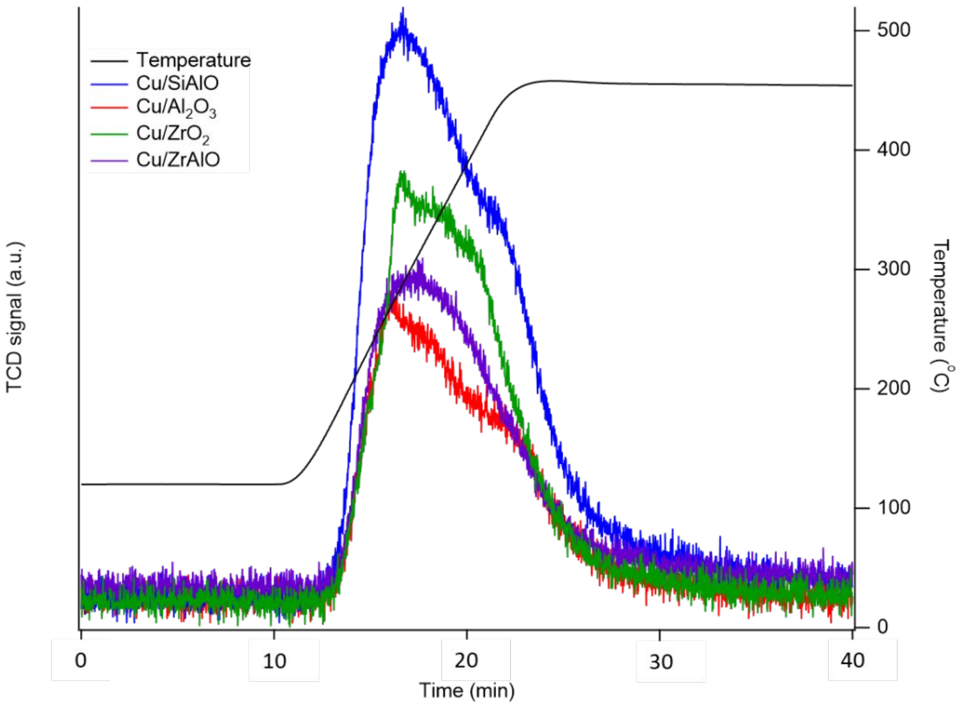

Figure S2. NH_3-TPD profiles of supported Cu catalysts.

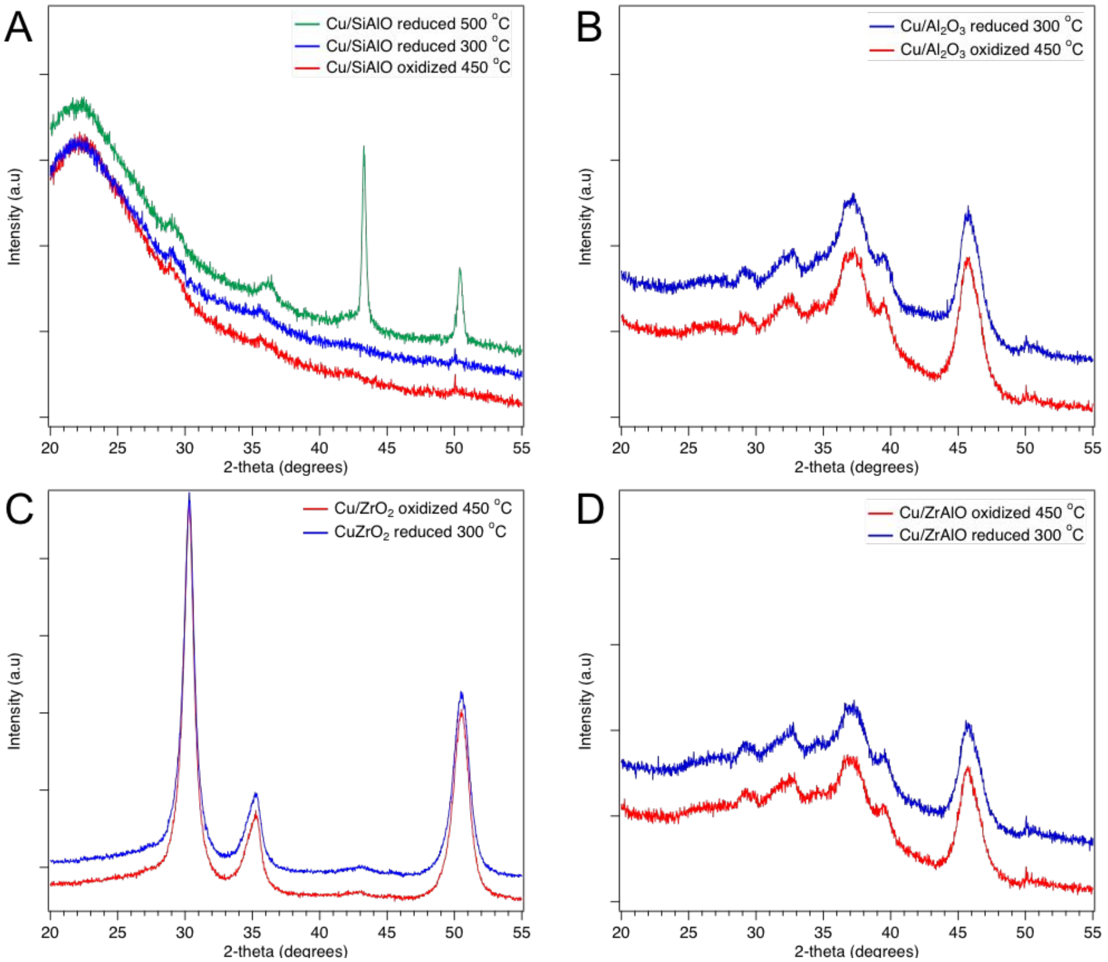

Figure S3. XRD patterns after oxidation and reduction for supported Cu catalysts (A) Cu/SiAlO, (B) Cu/Al_2O_3, (C) Cu/ZrO_2 and (D) Cu/ZrAlO.

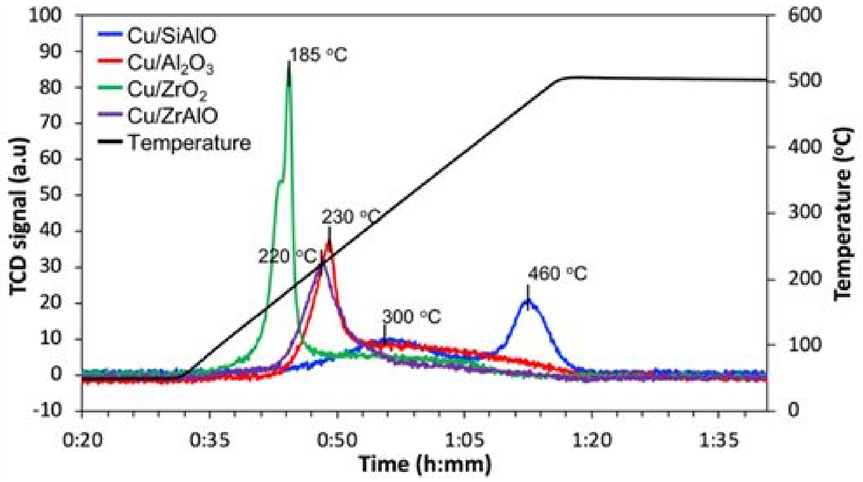

Figure S4. Temperature-programmed reduction profiles for supported Cu catalysts.

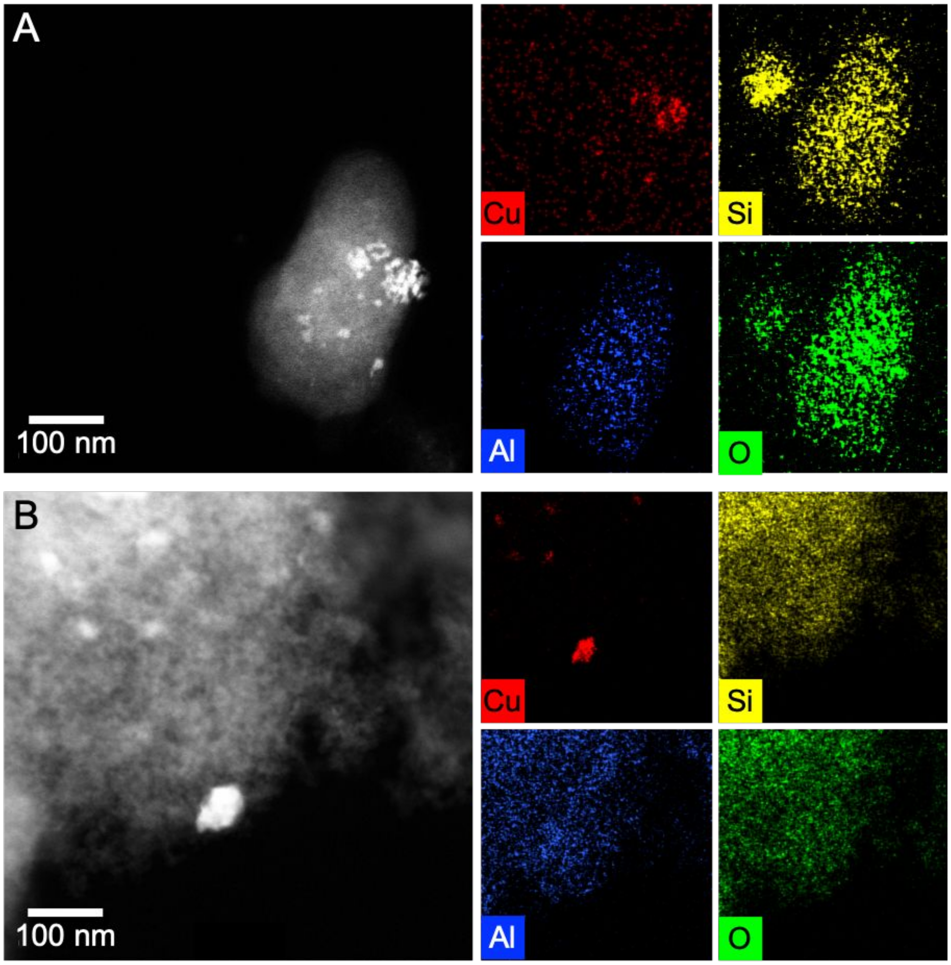

Figure S5. HAADF STEM images with EDS elemental maps of Cu/SiAlO after reduction in 5% H_2/N_2 at (A) 300 or (B) 450 °C, and passivation in 1% O_2/He.

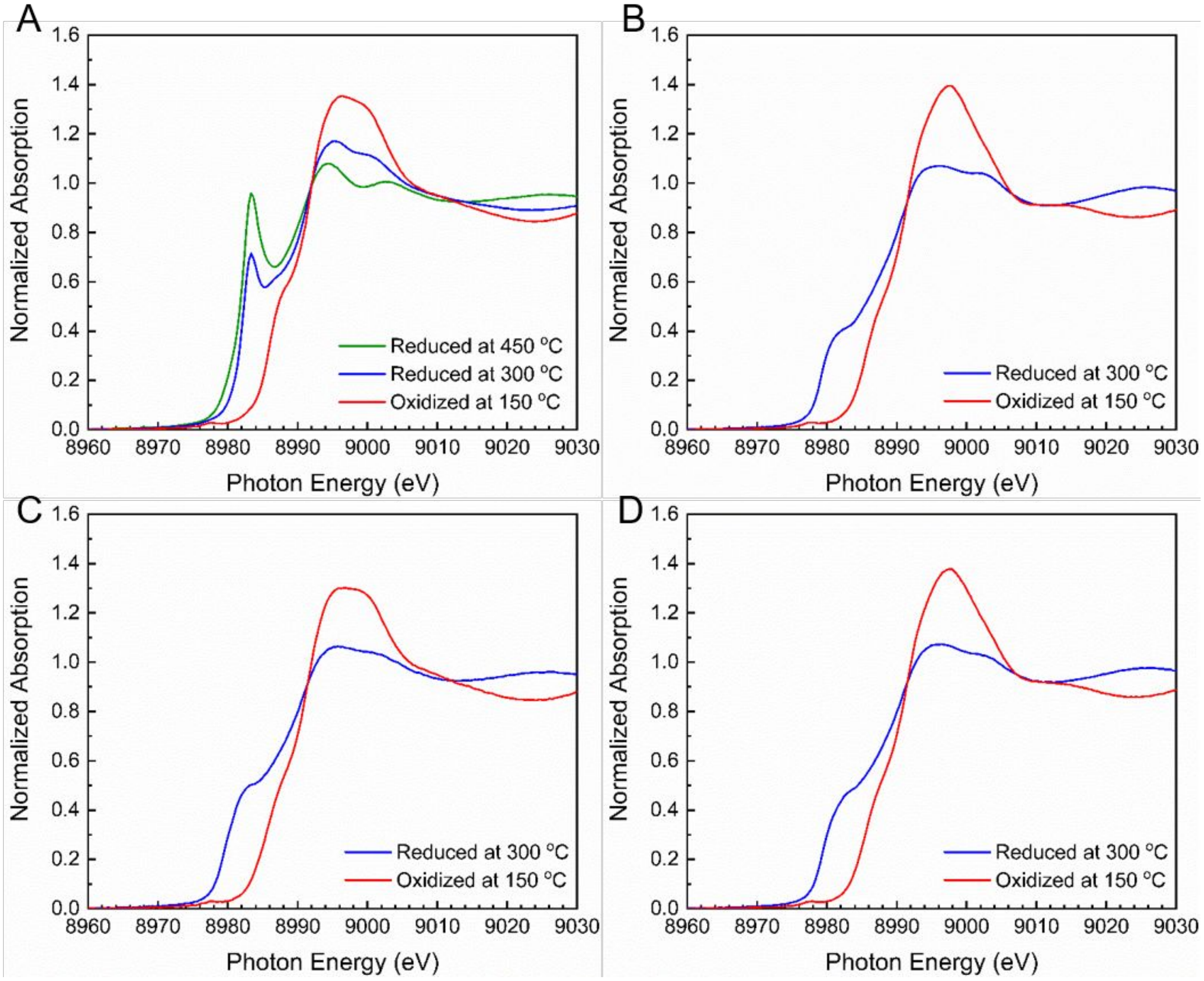

Figure S6. Normalized Cu K edge XANES after oxidation at 150 °C in air and reduction at 300 °C in H_2 for (A) Cu/SiAlO also reduced at 450 ºC, (B) Cu/Al_2O_3, (C) Cu/ZrO_2, and (D) Cu/ZrAlO.

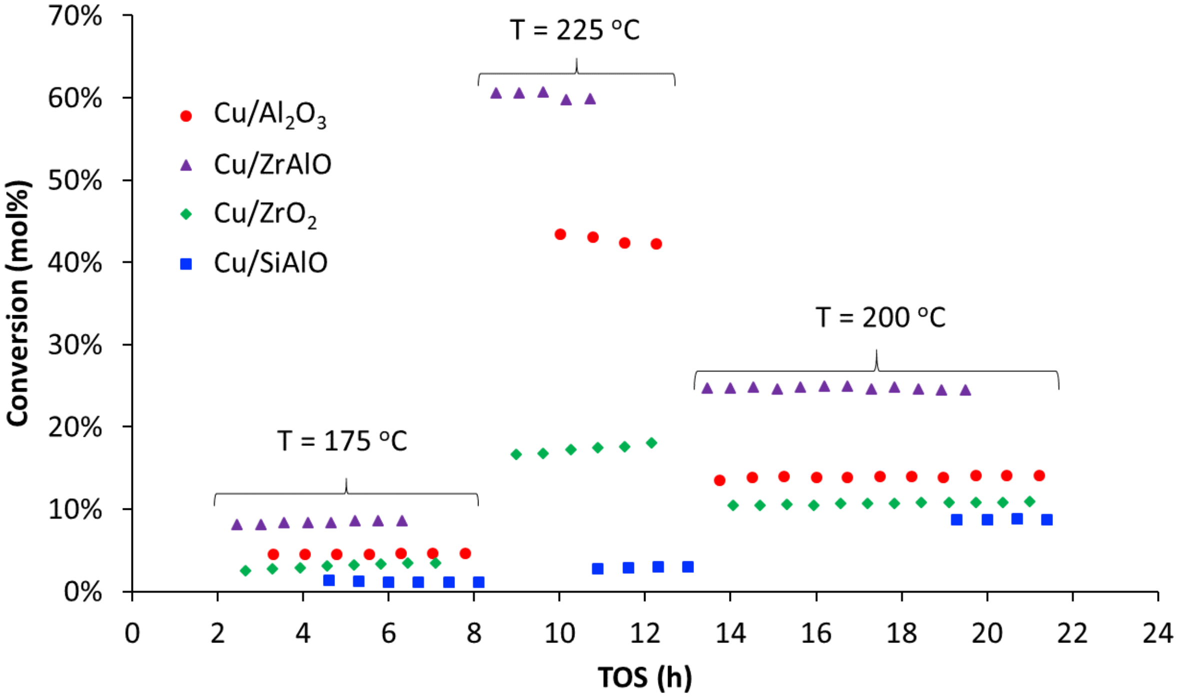

Figure S7. Methanol conversion versus time on stream (TOS) during the dehydrogenative coupling reaction over supported Cu catalysts at 175, 200 and 225 ^{o}C. Reaction conditions: 1.7 atm, WHSV = 5 h^{-1}. Cu catalysts were reduced with H_2 at 300 ^{o}C prior to reaction (450 ^{o}C for Cu/SiAlO)

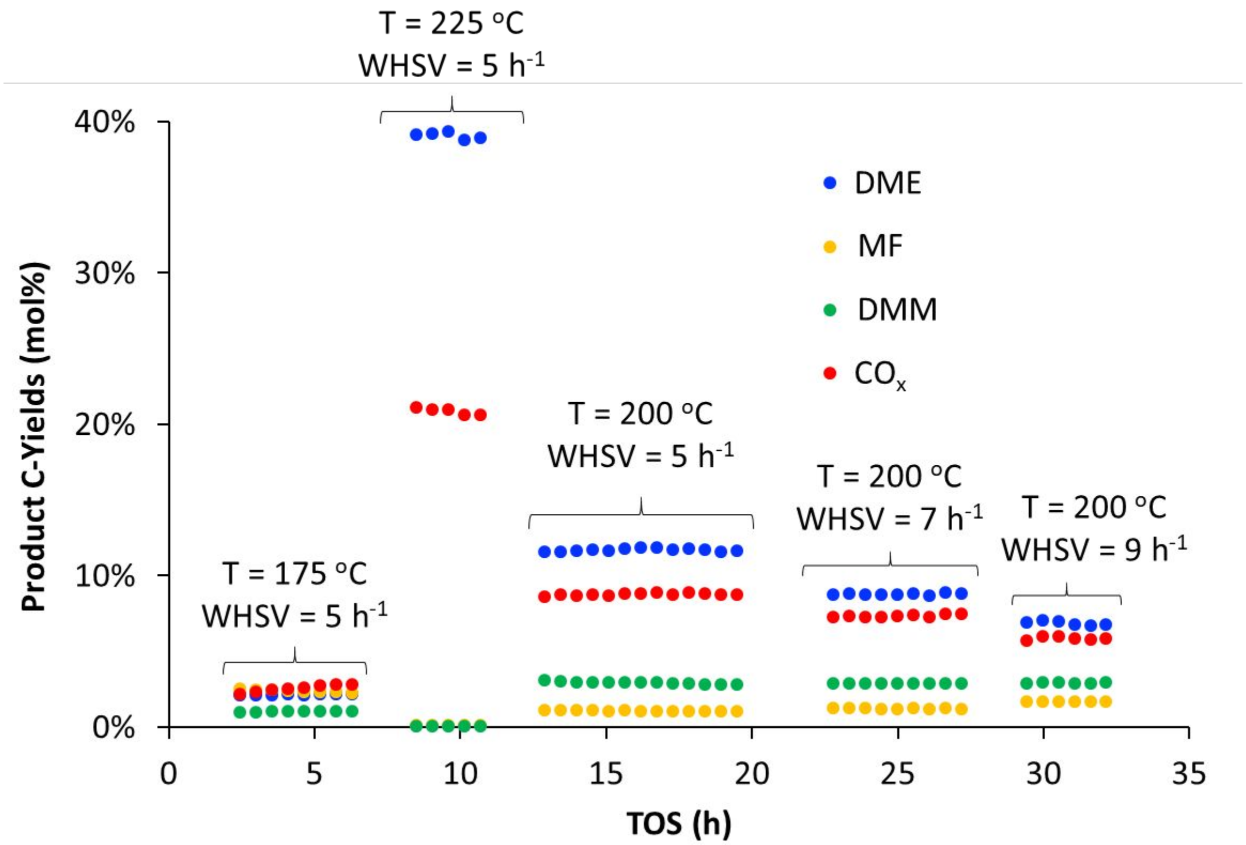

Figure S8. Plot of product yield versus time on stream (TOS) during the dehydrogenative coupling reaction of methanol over Cu/ZrAlO catalyst at 1.7 atm with varying temperature (T) and weight-hourly space velocity (WHSV).

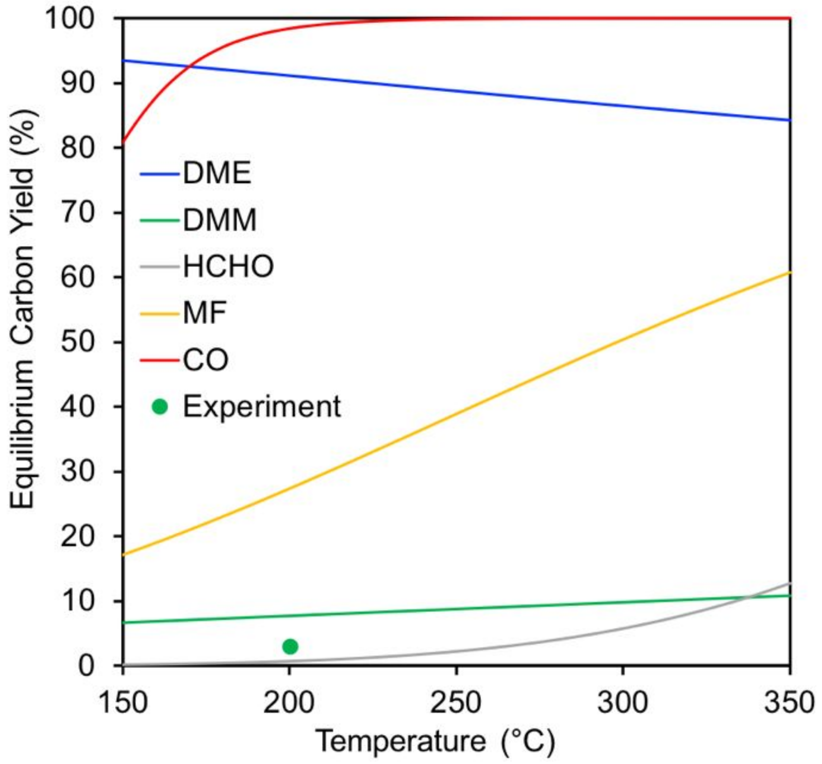

Figure S9. Equilibrium-limit C-yield for each product as a function of temperature for methanol conversion to dimethyl ether (DME), dimethoxymethane (DMM), formaldehyde (HCHO), methyl formate (MF), and carbon monoxide (CO). Experimental data point from the DHC reaction of methanol to DMM over the Cu/ZrAlO catalyst at 200 °C is included.

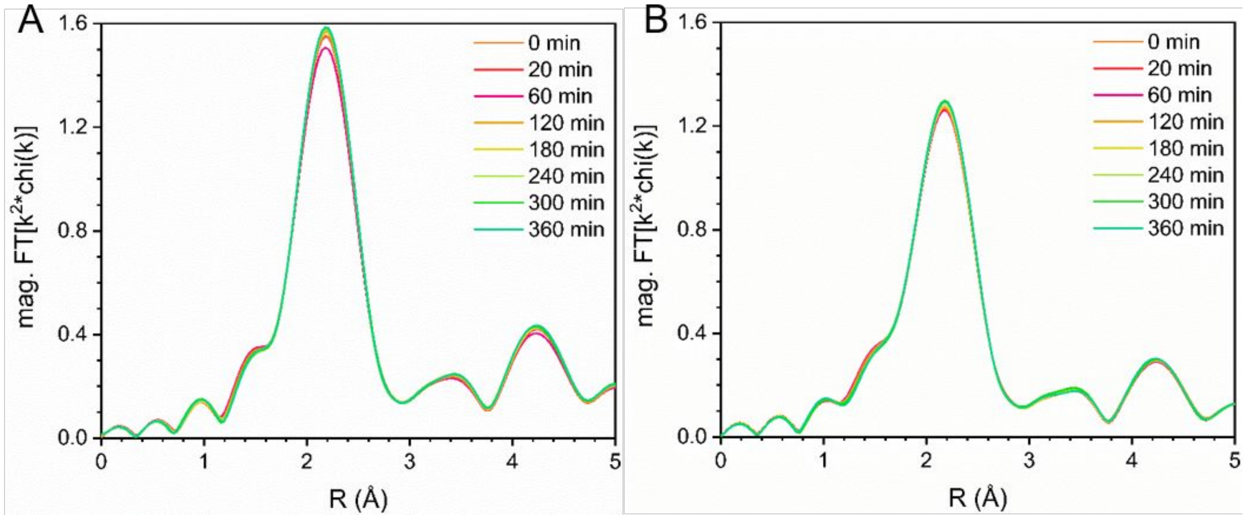

Figure S10. Magnitude of the Fourier transform of the Cu K edge k^2-weighted EXAFS of (A) Cu/Al_2O_3 and (B) Cu/ZrAlO at 200 °C in flowing methanol and helium mixture.

Table S1: Cu K edge EXAFS fitting parameters of oxide-supported Cu catalysts after oxidation at 150 °C and reduction at 300 °C

Catalyst	Treatment	Edge Energy (eV)	CN[1]	R (Å)	σ^2 (x10[3] Å[2])[2]	E_0 (eV)
**Cu/ZrAlO**	150 °C, 20% O_2/He	8985.9	3.7 ± 0.5 (Cu-O)	1.94 ± 0.01	5.8 ± 1.7	3.3 ± 1.7
	300 °C, 3.5% H_2/He	8979.3	1.0 ± 0.1 (Cu-O)<br>6.7 ± 0.8 (Cu-Cu)	1.88 ± 0.04<br>2.52 ± 0.01	5.8<br>11.4 ± 1.1	4.0 ± 5.6<br>2.7 ± 1.2
**Cu/Al_2O_3**	150 °C, 20% O_2/He	8985.5	3.6 ± 0.3 (Cu-O)	1.94 ± 0.01	5.8	3.7 ± 0.2
	300 °C, 3.5% H_2/He	8979.1	0.9 ± 0.2 (Cu-O)<br>7.8 ± 0.7 (Cu-Cu)	1.90 ± 0.04<br>2.53 ± 0.01	5.8<br>10.6 ± 0.9	5.1 ± 6.0<br>3.5 ± 1.0
**Cu/ZrO_2**	150 °C, 20% O_2/He	8985.6	3.9 ± 0.1 (Cu-O)	1.92 ± 0.01	5.8	1.4 ± 1.0
	300 °C, 3.5% H_2/He	8979.7	1.1 ± 0.3 (Cu-O)<br>5.5 ± 1.4 (Cu-Cu)	1.85 ± 0.06<br>2.51 ± 0.02	5.8<br>11.6 ± 2.4	2.6 ± 9.8<br>0.7 ± 2.6
**Cu/$SiAlO_x$**	150 °C, 20% O_2/He	8985.8	4.0 ± 0.3 (Cu-O)	1.94 ± 0.02	5.8	3.2 ± 1.2
	300 °C, 3.5% H_2/He	8982.2	2.7 ± 0.1 (Cu-O)	1.92 ± 0.01	5.8	5.3 ± 1.5
	450 °C, 3.5% H_2/He	8979.7	1.3 ± 0.2 (Cu-O)<br>3.0 ± 0.3 (Cu-Cu)	1.90 ± 0.04<br>2.53 ± 0.02	5.8<br>9.1 ± 2.7	8.6 ± 4.7<br>1.7 ± 3.1

[1] The amplitude reduction factor (S_0^2), determined to be 0.86 from a Cu foil, was held constant in the fit of the samples

[2] The fitted σ^2 value of 5.8 x 10^{-3} Å^2 determined from the Cu/ZrAlO was held constant for the other fits.

Table S2. Effect of reaction temperature on methanol conversion, product yields, and product selectivities over supported Cu catalysts. Catalysts were reduced with H_2 at 300 °C prior to reaction, except Cu/SiAlO which was reduced at 450 °C. X = conversion; Y_i = yield of product *i*; S_i = C-selectivity of product *i*. Reaction conditions: 1.7 atm, WHSV = 5 h^{-1}.

	Cu/Al_2O_3			Cu/SiAlO			Cu/ZrO_2			Cu/ZrAlO		
Temp. (°C)	*175*	*200*	*225*	*175*	*200*	*225*	*175*	*200*	*225*	*175*	*200*	*225*
X (%)	*4.6*	*14.0*	*42.8*	*1.9*	*2.4*	*5.5*	*3.2*	*10.7*	*17.3*	*8.4*	*24.7*	*61.3*
Y_{DME} (%)	2.5	10.1	35.3	0.4	1.4	4.3	0.0	0.1	0.1	2.1	11.7	39.7
Y_{MF} (%)	0.4	0.2	0.0	0.3	0.1	0.1	2.2	6.8	13.6	2.4	1.1	0.1
Y_{DMM} (%)	0.9	1.0	0.1	0.1	0.2	0.4	0.1	0.1	0.1	1.0	2.9	0.0
Y_{HCs} (%)	0.0	0.0	0.0	0.2	0.1	0.1	0.2	0.5	0.4	0.3	0.2	0.2
Y_{COx} (%)	0.7	2.7	7.4	0.9	0.6	0.6	0.7	3.3	3.1	2.6	8.8	21.3
S_{DME} (%)	*54.6*	72.3	82.5	18.9	57.7	77.8	0.7	0.5	0.8	25.6	47.4	64.7
S_{MF} (%)	9.6	1.3	0.1	13.9	3.1	2.3	67.9	63.5	78.7	28.5	4.4	0.2
S_{DMM} (%)	19.0	7.0	0.2	6.8	8.0	7.1	2.0	0.7	0.4	12.4	12.0	0.1
S_{HCs} (%)	1.0	0.2	0.0	11.4	3.7	1.8	7.2	4.6	2.5	3.0	0.8	0.3
S_{COx} (%)	15.9	19.3	17.2	49.0	27.6	11.0	22.2	30.7	17.6	30.5	35.4	34.7

Table S3: Cu K-edge EXAFS fitting parameters of Cu/ZrAlO at 200 °C under flowing methanol/helium

Time (min)	CN: Cu-Cu	R (Å)	σ^2 (x10^3 Å^2)	E_0 (eV)
**0**	8.2 ± 0.5	2.51 ± 0.01	14.2 ± 0.5	2.4 ± 1.2
**20**	8.3 ± 0.5	2.51 ± 0.01	14.2 ± 0.5	2.3 ± 1.1
**60**	8.3 ± 0.5	2.51 ± 0.01	14.2 ± 0.5	2.3 ± 1.1
**120**	8.3 ± 0.5	2.51 ± 0.01	14.2 ± 0.5	2.4 ± 1.2
**180**	8.3 ± 0.5	2.51 ± 0.01	14.2 ± 0.5	2.4 ± 1.1
**240**	8.4 ± 0.5	2.51 ± 0.01	14.2 ± 0.5	2.4 ± 1.2
**300**	8.4 ± 0.5	2.51 ± 0.01	14.2 ± 0.5	2.5 ± 1.1
**360**	8.4 ± 0.5	2.51 ± 0.01	14.2 ± 0.5	2.5 ± 1.1

Table S4: Cu K-edge EXAFS fitting parameters of Cu/Al_2O_3 at 200 °C under flowing methanol/helium

Time (min)	CN: Cu-Cu	R (Å)	σ^2 (x10^3 Å^2)	E_0 (eV)
**0**	9.4 ± 0.6	2.52 ± 0.01	13.6 ± 0.4	2.5 ± 1.2
**20**	9.6 ± 0.5	2.52 ± 0.01	13.6 ± 0.4	2.6 ± 1.0
**60**	9.6 ± 0.6	2.52 ± 0.01	13.6 ± 0.4	2.6 ± 1.1
**120**	9.8 ± 0.6	2.52 ± 0.01	13.6 ± 0.4	2.6 ± 1.1
**180**	9.7 ± 0.6	2.52 ± 0.01	13.6 ± 0.4	2.7 ± 1.1
**240**	9.7 ± 0.6	2.52 ± 0.01	13.6 ± 0.4	2.7 ± 1.1
**300**	9.8 ± 0.5	2.52 ± 0.01	13.6 ± 0.4	2.7 ± 1.0
**360**	9.8 ± 0.6	2.52 ± 0.01	13.6 ± 0.4	2.7 ± 1.1

References

1. Zhang, J.; Fang, D.; Liu, D. Evaluation of Zr–Alumina in Production of Polyoxymethylene Dimethyl Ethers from Methanol and Formaldehyde: Performance Tests and Kinetic Investigations. *Ind. Eng. Chem. Res.* **2014,** *53* (35), 13589-13597.
2. Emeis, C. A. Determination of Integrated Molar Extinction Coefficients for Infrared Absorption Bands of Pyridine Adsorbed on Solid Acid Catalysts. *J. Catal.* **1993,** *141* (2), 347-354.
3. Maier, S. M.; Jentys, A.; Lercher, J. A. Steaming of Zeolite BEA and Its Effect on Acidity: A Comparative NMR and IR Spectroscopic Study. *J. Phys. Chem. C* **2011,** *115* (16), 8005-8013.

4. Ravel, B.; Newville, M. ATHENA, ARTEMIS, HEPHAESTUS: Data Analysis for X-ray Absorption Spectroscopy using IFEFFIT. *J. Synchrotron Radiat.* **2005,** *12* (Part 4), 537-541.

5. Rehr, J. J.; Mustre de Leon, J.; Zabinsky, S. I.; Albers, R. C. Theoretical X-ray Absorption Fine Structure Standards. *J. Am. Chem. Soc.* **1991,** *113* (14), 5135-5140.

6. Fogler, H. S. Diffusion and Reaction in Porous Catalysts. In *Elements of Chemical Reaction Engineering,* 4th ed.; Prentice Hall, 2006; pp 15-1-48.

7. Fogler, H. S. Mass Transfer Limitations in Reacting Systems. In *Elements of Chemical Reaction Engineering,* 4th ed.; Prentice Hall, 2006; pp 14-1-44.
